# Decolonizing the Narrative of Indigenous Food Sovereignty: A Scoping Review

**DOI:** 10.1016/j.cdnut.2026.109430

**Published:** 2026-07-02

**Authors:** Ines Sebai, Gabriela Berron Cadenas, Chris Furgal, Alejandro Argumedo, Treena Delormier

**Affiliations:** 1 School of Human Nutrition, McGill University, Montréal, QC, Canada; 2 School of Medicine and Health Sciences TecSalud, Monterrey Institute of Technology and Higher Education (Tecnológico de Monterrey), Monterrey, Mexico; 3 Indigenous Environmental Studies and Sciences, Trent University, Peterborough, ON, Canada; 4 Swift Foundation and Asociacion ANDES, Cusco, Peru

**Keywords:** Indigenous food sovereignty, Indigenous peoples, food sovereignty, traditional food systems, scoping review

## Abstract

This scoping review explores Indigenous food sovereignty (IFS) from an Indigenous perspective as a decolonizing framework, grounded in the cultural, spiritual, and ecological values of Indigenous communities. IFS integrates self-determination, cultural continuity, and ecological stewardship, offering a holistic approach for Indigenous communities to reclaim control over their food systems. By synthesizing existing literature, this review identifies core principles of IFS, including community governance, the revitalization of traditional practices, and respectful relationships with the environment. Examples from the literature highlight consistent themes across diverse Indigenous contexts, such as community gardens, traditional hunting and gathering, and intergenerational knowledge sharing. These practices illustrate Indigenous communities’ adaptation of food sovereignty principles to contemporary challenges, fostering cultural renewal, environmental sustainability, and community resilience. Additionally, this article reviews tools for assessing food sovereignty, addressing challenges in applying these tools across varied settings, and their potential to support decolonizing efforts. The findings underscore the need for policy advocacy and the recognition and support of Indigenous rights, including land restoration, the protection of traditional knowledge, and resources for sustainable food production. This work contributes to the broader discourse on Indigenous knowledge, cultural preservation, and food security, promoting a more inclusive and sustainable global food system.

## Introduction

Indigenous peoples, according to the United Nations, encompass >476 million individuals across >90 countries, representing a rich diversity of cultures, languages, and traditions [1]. Although there is no single, universally adopted definition, Indigenous peoples are commonly recognized as communities that have historical continuity with precolonial and/or presettler societies, maintain distinct cultural practices, languages, and social structures, and have a deep-seated connection to their lands and natural resources [1,2]. However, the political recognition of this definition can vary significantly across regions. The term tends to be more clearly applied in settler-colonial contexts such as Canada, Australia, and New Zealand, whereas in other parts of the world, including Africa and Asia, defining Indigeneity is often more contested because of differing historical and political dynamics. In this context, self-identification remains a key criterion for defining Indigenous status, as stipulated by the United Nations. Indigenous peoples worldwide, including groups such as the Inuit, Saami, Maori, and First Nations in Canada, often form nondominant segments within national populations and face unique challenges in safeguarding their cultural and land rights [1].

Various countries, such as Canada, Australia, and New Zealand, have recognized Indigenous peoples within their nation-state legal frameworks. These countries have developed policies acknowledging the distinct rights of Indigenous communities to preserve their cultural heritage and manage their lands. Canada, e.g., formally recognizes the unique legal status of First Nations, Métis, and Inuit peoples within its Constitution Act, 1982, Section 35, which grants certain rights related to land, culture, and self-governance [3]. Similarly, New Zealand has established the Treaty of Waitangi as a foundational document that defines the rights and responsibilities between the Māori and the Crown [4]. These national frameworks, although important, often conflict with international frameworks, especially where greater recognition is given to Indigenous assertion of self-determination pertaining to such things as land, resources, identity, language, and other aspects of daily life.

At the international level, the United Nations Declaration on the Rights of Indigenous Peoples (UNDRIP), adopted in 2007, provides a comprehensive framework affirming the rights of Indigenous peoples to self-determination, cultural preservation, and land sovereignty. Article 3 of UNDRIP asserts Indigenous peoples’ right to self-determination, allowing them to freely pursue their social, economic, and cultural development [5]. This principle of self-determination is foundational because it supports Indigenous peoples in maintaining autonomous control over their governance, cultural practices, and resource management. Furthermore, Articles 25 through 28 address Indigenous communities’ rights to their lands, territories, and resources, recognizing these as central to their cultural identity and sustainability. For Indigenous communities, these rights are not merely economic but are intertwined with spiritual beliefs, social structures, and identity. The right to freely practice and protect these connections to land is essential for the survival and well-being of Indigenous peoples and cultures [5].

In conjunction with UNDRIP, the United Nations Declaration on the Rights of Peasants and Other People Working in Rural Areas (UNDROP)—which defines “peasants” broadly as small-scale farmers, agricultural workers, and rural populations engaged in food production—complements Indigenous rights by addressing food sovereignty specifically. Adopted in 2018, UNDROP enshrines the rights of rural populations, including Indigenous communities, to determine their own food systems and agricultural practices [6]. This declaration reflects a broader commitment to food sovereignty, which is described as the right of people to access healthy and culturally appropriate food produced through ecologically sound and sustainable methods. Key articles in UNDROP, such as Article 5 on the right to natural resources, Article 15 on the right to produce food and the right to adequate nutrition, Article 17 on the right to land, and Article 19 on the right to seeds, emphasize the rights of rural populations to participate in decision-making processes concerning food production and to secure access to essential resources such as land, seeds, and water [6]. By supporting the autonomy of rural and Indigenous communities in food-related decision-making, UNDROP fosters resilience in food systems, promotes environmental stewardship, and safeguards cultural values embedded within these practices. However, it does not center the distinct Indigenous rights of Indigenous peoples, which are foundational to Indigenous food sovereignty (IFS). Although it may be argued that the intersection of UNDRIP and UNDROP lays a foundational framework for IFS, it does not explicitly recognize Indigenous peoples but rather includes them as others alongside peasant and rural peoples. However, IFS is best understood from Indigenous perspectives and experiences.

The concept of Indigenous Peoples’ food sovereignty has gained increasing attention in academic literature and community practice as a response to colonial structures, systems, and impacts that disrupt traditional ways of life and continue to impose control over Indigenous lands and resources. Colonialism has displaced Indigenous communities from their ancestral territories, disrupted their foodways, and replaced them with systems designed to serve colonial interests. Although it draws from international rights frameworks such as UNDRIP and UNDROP, Indigenous Peoples’ food sovereignty is rooted in cultural, spiritual, and ecological values specific to Indigenous worldviews. However, despite its growing use, the concept is described and defined in various ways, differs across regions, and is sometimes conflated with food security. As such, a clearer synthesis of IFS—grounded in Indigenous perspectives—is needed to support a more comprehensive understanding of the concept and to help guide both research and policy.

### Objectives of the review

This scoping review aimed to synthesize the existing literature on IFS, presenting the concept as articulated from Indigenous perspectives—particularly those grounded in Indigenous authorship, community-based research, and the inclusion of Indigenous worldviews—and highlighting its significance beyond food access. In doing so, it addresses an important gap in the literature by bringing together definitions and key principles into a single conceptual synthesis.

Although existing studies have primarily focused on interventions, outcomes, and assessment tools, this review instead examines how IFS is defined and understood across the literature. It further seeks to identify key elements and principles—particularly cultural, spiritual, and ecological dimensions—and to trace the framing of the movement within Indigenous contexts. By providing a structured synthesis of definitions and key principles grounded in Indigenous perspectives, this review contributes to clarifying the conceptual foundations of IFS and offers a framework that may inform future research, policy, and practice. Finally, where available, the review includes practical examples and considers tools used to assess IFS, as reported within the included studies.

## Methods

### Study design

**FIGURE 1:**
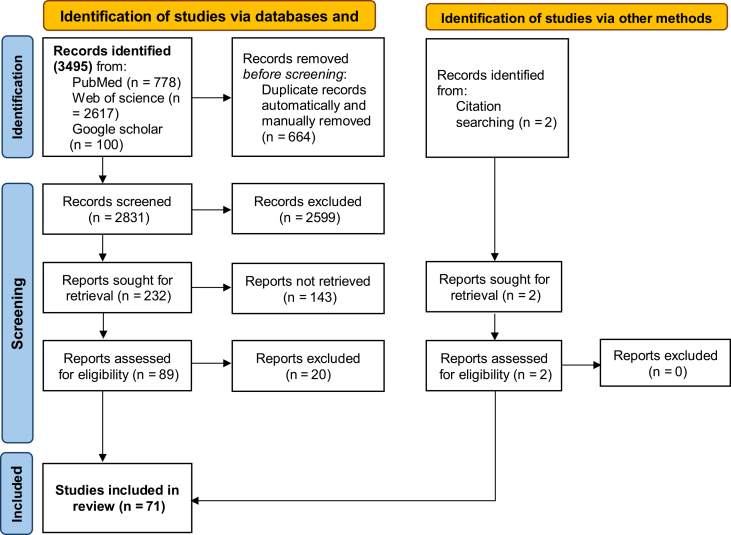
PRISMA 2020 flow diagram of the search strategy and study selection.

This scoping review followed the PRISMA extension for Scoping Reviews (ScR) guidelines [7], alongside Arksey and O’Malley’s framework [8]. This framework included identifying the research question, identifying relevant studies, study selection, charting the data, collating, summarizing, and reporting the findings. This structured approach is ideal for exploring the broad, complex, and varied landscape of IFS, mapping the existing knowledge, identifying gaps, and synthesizing key constructs. The review protocol was preregistered on the Open Science Framework (OSF). We also adhered to PRISMA-ScR 2020 guidelines to ensure transparency, replicability, and thoroughness in data identification, screening, and inclusion (PRISMA 2020 flow diagram, Figure 1).

### Search strategy

A structured search of peer-reviewed literature was conducted across 3 main databases—PubMed, Web of Science, and Google Scholar—in October 2024. These databases were selected for their broad coverage, each offering unique contributions: PubMed provides extensive access to health-related research, essential due to the connections between IFS and health; Web of Science offers multidisciplinary coverage for a broad perspective; and Google Scholar was used to complement database searches and identify potentially relevant articles, including gray literature in the form of report, which may not have been captured in other databases and may provide additional insights into IFS practices and policies. Only peer-reviewed articles and selected reports published in English or French were included in this review, which may have limited the representation of studies from other linguistic and regional contexts.

### Search terms

The search terms used were “Indigenous food sovereignty” and “Indigenous food security,” connected by the Boolean operator “OR.” This broad search aimed to capture a wide range of literature up to October 2024, without applying additional filters or geographic restrictions, to ensure inclusivity in the studies retrieved across diverse global contexts.

### Selecting sources of evidence (inclusion/exclusion criteria)

#### Inclusion criteria

Articles were included if they were published in English or French (the languages spoken by the primary reviewer, IS) and addressed the research objectives, specifically focusing on IFS. Studies were selected based on their provision of description, principles, or practices related to IFS.

#### Exclusion criteria

Exclusions were made for studies that did not report findings specific to Indigenous peoples, focused on non-Indigenous populations or topics unrelated to IFS, or were full books (although individual chapters aligning with review objectives were considered). Articles lacking specificity regarding Indigenous practices, tools, and sovereignty concepts were also excluded.

During the eligibility screening, each article’s potential to address the objectives of the review was assessed based on its ability to fill relevant fields within a standardized data chart. An article’s relevance was not based on its ability to complete all chart fields; rather, relevance was determined by its alignment with the primary review objectives. Specifically, this review focused on definitions and key elements of IFS, with practical examples and assessment tools considered as secondary elements when reported within studies addressing the primary objectives. Articles that did not address the core concepts or key elements of IFS were excluded because their inclusion would not support the primary purpose of this review.

### Study selection

The study selection process involved several stages to ensure thoroughness and relevance:

1) Initial screening: Titles and abstracts were initially screened by the primary reviewer (IS) to assess their relevance based on the inclusion and exclusion criteria. Articles with ambiguous abstracts were retained for full-text review to avoid prematurely excluding potentially relevant studies.
2) Secondary screening: Articles passing the first round underwent a second screening, focusing explicitly on “IFS” to further refine the selection. At this stage, articles addressing only Indigenous food security without reference to IFS were excluded because they did not align with the primary objectives of the review.
3) Full-text review: Full-text screening was conducted collaboratively by IS and GBC. Both authors independently evaluated each article’s content to determine alignment with all inclusion criteria. Disagreements were resolved through discussion, and the final list of included articles was validated by all coauthors. This process ensured that all studies meeting the inclusion criteria were included in the review.
4) Citation screening: Reference lists of relevant studies were manually reviewed to identify additional studies not captured in the initial database searches, allowing the inclusion of supplementary relevant sources.

### Charting the data (data extraction and synthesis)

Data extraction and synthesis were conducted using a standardized data chart to ensure consistency and facilitate comprehensive analysis across studies. Key elements documented for each article included the following information:

1) Author and year: Basic identifying information was recorded to track sources and publication dates.
2) Study design: Each study’s methodological approach (e.g., systematic review, case study, and commentary) was noted to assess the range and rigor of research approaches in the literature.
3) Community and region: Information about the specific Indigenous communities and geographical regions involved in each study was recorded to capture diversity and contextual factors that shape food sovereignty practices.
4) Definitions of IFS: Descriptions were carefully documented, noting any unique interpretations or emphases to understand how IFS is conceptualized within different studies.
5) Key elements of food sovereignty: Foundational components, such as cultural, spiritual, and ecological aspects, were recorded to identify the main principles that define IFS.
6) Indigenous perspectives and research approaches: Particular attention was given during data extraction to identifying Indigenous authorship (e.g., through author self-identification), the inclusion of Indigenous perspectives (such as direct quotations or community voices), and the use of community-based participatory research (CBPR) approaches. These elements were documented and considered during the synthesis to ensure that Indigenous perspectives were meaningfully reflected in the analysis.
7) Tools to assess food sovereignty: Any assessment tools reported within the included studies were documented, including their applicability, effectiveness, and limitations within Indigenous contexts.
8) Practical examples and daily practices: Examples illustrating IFS in action were recorded from studies that met the primary inclusion criteria, namely those addressing definitions and/or key elements of IFS. Once a study was included, any relevant examples reported within that study were also extracted to provide real-world context and showcase the diversity of Indigenous approaches to food sovereignty.

The data synthesis identified overarching themes and trends through an inductive thematic approach, contributing to a comprehensive understanding of components, challenges, and current assessment methods related to IFS. Extracted data were reviewed and grouped based on recurring concepts and patterns identified across multiple studies, with themes developed iteratively by identifying similarities and differences in how IFS was described in the literature. The synthesis was conducted by the primary author (I.S.), with support from the second coauthor (G.B.C.), and was reviewed and approved by all coauthors. This thematic synthesis aimed to map the existing knowledge by organizing and synthesizing concepts and findings across studies into thematic categories, highlighting research needs and informing policy support.

### Author positionality

This review is informed by decolonizing approaches to research, which emphasize relationality, reflexivity, and the centering of Indigenous knowledge systems. In this context, the synthesis prioritizes Indigenous authorship, community-based research, and interpretations grounded in Indigenous perspectives, while acknowledging the limitations of working within academic and peer-reviewed frameworks.

The authors acknowledge that this review is conducted from an academic perspective and recognize the importance of situating IFS within Indigenous worldviews and lived experiences. The perspectives presented in this manuscript are informed by a collaborative team of Indigenous and non-Indigenous researchers, guided by principles of respect for Indigenous knowledge systems and relational accountability. Although efforts were made to prioritize and highlight Indigenous perspectives, the authors recognize that the findings are shaped by their roles as researchers and the limitations of working primarily with peer-reviewed literature. This review aimed to respectfully synthesize existing knowledge while acknowledging the importance of Indigenous-led research and knowledge systems in advancing the field.

## Results and Discussion

The initial search yielded 3495 records across 3 databases: 778 from PubMed, 2617 from Web of Science, and 100 from Google Scholar (the first 10 pages). Duplicate records were removed in EndNote (Clarivate, Philadelphia, PA, USA), both automatically and manually, before uploading the remaining records to Covidence (Veritas Health Innovation, Melbourne, VIC, Australia) for further management. After deduplication in Covidence, 2860 unique records remained, with an additional 27 duplicates removed. From these, 2831 records were selected for screening based on their titles and abstracts to assess relevance. See Figure 1 for the PRISMA flow diagram detailing each stage of the screening and selection process.

In the first round of title and abstract screening, 2599 records were excluded, leaving 230 papers for further consideration. During this stage, we observed that many articles focused exclusively on Indigenous food security without addressing the broader dimensions of IFS. Although both concepts were initially included in the search strategy, preliminary analysis revealed that Indigenous food security was often framed within conventional paradigms focused on food access and availability. In contrast, IFS was more aligned with self-determined, culturally grounded food systems. To enable a more precise and in-depth exploration of these dimensions, we narrowed the scope of the review to include only articles that directly addressed IFS. This resulted in 89 articles for full-text review. In addition, during full-text review, reference lists of all studies were screened, through which 2 additional articles were identified and included in the review. After reviewing these full texts, 71 articles were retained for final inclusion, whereas 20 were excluded for not aligning with the study’s objectives.

### Characteristics of included papers

The final 71 articles represent a broad range of conceptual constructs, methodologies, regional foci, and thematic insights into IFS (Supplementary Table 1 for the included studies). The majority of studies used to examine this topic employ qualitative methods, including case studies and CBPR, to capture community-specific perspectives. Indigenous methodologies are prominent, with many studies prioritizing relational and participatory approaches that center Indigenous voices.

Geographically, the studies cover Indigenous communities primarily in North America, with additional representation from South America and Australia. This distribution highlights the growing interest in the concept of Indigenous peoples’ food sovereignty across diverse regions and reflects the broader reach of the movement.

## Section 1: Definitions of IFS

This section presents a synthesis of the definitions, evolution, and conceptual framing of IFS identified across the included studies.

### Evolution of the concept of food sovereignty

The concept of food sovereignty originated in the early 1990s in response to global agricultural policies and neoliberal trade practices that prioritized large-scale, industrial agriculture over local, community-driven food production systems. These industrial food systems and economies marginalized small-scale farmers by reducing traditional food systems and increasing dependency on heavily processed, imported foods. In 1993, La Vía Campesina, an international movement of peasants and small-scale farmers, introduced the term “food sovereignty,” initially defining it as

“The right of people to have access to healthy and culturally appropriate foods, while defining their own food systems.”

This initial definition emphasized the rights of marginalized communities, especially those adversely affected by the global food systems shaped by colonial and neoliberal structures, to reclaim control over their local food systems, prioritize food security, and resist reliance on imported, often heavily processed foods.

In the years that followed, food sovereignty became a guiding principle for communities worldwide who sought to challenge the dominance of corporate-controlled food systems that disrupted local foodways and diminished traditional agricultural practices. The movement gained momentum through declarations such as the 2007 Nyéléni Declaration, produced at the World Forum for Food Sovereignty in Sélingué, Mali, by a global coalition of >500 representatives from >80 countries, including peasants, Indigenous peoples, and other food system actors. This declaration further solidified the call for community-based control over food systems and recognized the integral role of cultural and ecological values in sustainable food practices.

“Food sovereignty is the right of peoples to healthy and culturally appropriate food produced through ecologically sound and sustainable methods, and their right to define their own food and agriculture systems. It puts those who produce, distribute and consume food at the heart of food systems and policies rather than the demands of markets and corporations. It defends the interests and inclusion of the next generation. It offers a strategy to resist and dismantle the current corporate trade and food regime, and directions for food, farming, pastoral and fisheries systems determined by local producers. Food sovereignty prioritizes local and national economies and markets and empowers peasant and family farmer-driven agriculture, artisanal fishing, pastoralist-led grazing, and food production, distribution and consumption based on environmental, social and economic sustainability.” (Nyéléni Declaration)

In Indigenous communities, food sovereignty has always been embedded in longstanding food systems, cultural responsibilities, and relationships to land [9]. The articulation of IFS emerged as a way to affirm these holistic frameworks and to distinguish them from broader movements. IFS incorporates dimensions beyond agricultural autonomy, including cultural identity, spiritual relationships, ecological stewardship, and self-determination [10,11].

### IFS: expanding the framework

What is now articulated as IFS reflects longstanding food systems, cultural responsibilities, and relationships to land that Indigenous peoples have always practiced. Although the broader food sovereignty movement helped bring visibility to issues of local food control, the concept of IFS emerged to affirm Indigenous worldviews and to highlight distinct dimensions rooted in Indigenous rights, identities, and holistic relationships with food systems [12]. Indigenous perspectives view food not merely as a resource but as a sacred, interconnected element within an ecological and social framework that fosters community resilience and cultural identity [13].

In the context of IFS, Indigenous authors such as Morrison [9] and other scholars argue that the concept is best described rather than strictly defined, as definitions can impose limitations that overlook the diversity of Indigenous experiences and practices. This perspective emphasizes that IFS should be interpreted and adapted by individual communities, allowing the concept to shift according to each community’s specific needs, contexts, and cultural practices [9,[14], [15], [16], [17], [18], [19], [20], [21], [22], [23]]. In this sense, IFS constructs remain fluid, allowing the concept the flexibility required to address Indigenous peoples’ distinct ecological and social settings [24]. The variation in definitions of IFS across studies reflects the diversity of perspectives and contexts within Indigenous communities. Rather than indicating conceptual inconsistency, this variation may be understood as a reflection of the Nation-specific and place-based nature of Indigenous food systems. IFS is therefore not a singular or fixed concept but one shaped by distinct cultural, ecological, and governance contexts. As such, differences in how it is defined and articulated across studies highlight the importance of understanding IFS as a contextually grounded and locally defined concept, rooted in the lived realities and knowledge systems of Indigenous peoples.

This adaptability is evident across multiple descriptions of IFS in the literature (Supplemental Table 1), each contributing to a nuanced understanding of the concept. Morrison [9] describes IFS as “*a restorative framework for health and community development that appreciates the ways in which we can work together cross-culturally to heal our relationships with one another and the land, plants, and animals that provide us with our food,*” emphasizing that IFS encompasses more than just food access. It integrates principles of sacred or divine sovereignty, self-determination, participation, and legislative and policy support [9]. This restorative framing resonates across diverse Indigenous contexts where food sovereignty efforts are deeply tied to healing from the ongoing impacts of colonialism [25]. Legal and political recognition of Indigenous rights varies globally, yet in many cases, such recognition remains shaped by colonial frameworks that have historically—and often continue to—undermine Indigenous governance and well-being. As such, IFS must also be understood as a part of broader processes of healing, resistance, and reconciliation [26,27]. In Canada, for instance, Indigenous rights are constitutionally recognized, and the Truth and Reconciliation Commission has emphasized the importance of building respectful relationships between Indigenous peoples and settlers as a part of a decolonial and socially just future.

Stiegman and Pictou [28] underscore that IFS is framed through the Mi’kmaq legal concept of *Netukulimk*, which is described as a relational and ethical approach to resource use that provides for the health and well-being of the community while ensuring the integrity of ecosystems for future generations. This concept aligns with the broader Mi’kmaq worldview of maintaining right relations with land, water, and nonhuman kin. It includes a deep spiritual connection to food and the land, framing IFS as a system that nurtures community well-being through culturally relevant practices, intergenerational knowledge sharing, and environmental balance [28].

“Indigenizing food sovereignty in Mi’kma’ki entails centering Netukulimk, a Mi’kmaw legal concept relating to providing for the health and wellbeing of communities and future generations, by learning how to live well with the Land and Waters. Efforts to rebuild and to strengthen Mi’kmaq Food Sovereignty are not just about accessing traditional foods; they are about maintaining reciprocal relations with non-human kin including plants and animal relatives that are sources of food. These in turn, are part of larger efforts by Mi’kmaq communities to re-invigorate the Land and Water-based practices and the worldview and knowledge system rooted in those practices; as well as the governance structures and political capacity to enact these (Pictou 2019; Prosper et al. 2011) [28].”

Similarly, Cidro et al. [25] describe IFS as rooted in sacred responsibilities with the land and all living beings, positioning it as an adaptable process aligned with each community’s ecological and cultural realities. Maudrie et al. [10] expand on this by defining IFS as a holistic approach to food, emphasizing relationality, reciprocity, and values distinct from those of dominant society. They highlight that IFS is often misunderstood as merely a solution to food security, rather than an entirely different food system predicated on Indigenous values and decolonization.

“Indigenous Food Sovereignty (IFS) is a holistic approach to food that incorporates values of relationality, reciprocity, and relationships. Fundamental differences exist between food security and food sovereignty, yet dominant society often reduces IFS as a solution to food security, rather than an entirely different food system that is predicated on values that contrast with that of dominant society.” [10]

The overall concept of IFS is characterized by its fluidity and adaptability. It is recognized as a framework, a movement, and an ongoing process grounded in cultural responsibilities, relational worldviews, and the pursuit of self-determined futures [21,29,30]. IFS reflects the understanding that each Indigenous community or Nation must define it in relation to their own ecological and cultural context, historical experience, and aspirations. Indigenous perspectives emphasize that food sovereignty is not static but continually shaped through the reclaiming and revitalization of traditional practices and knowledge systems that sustain both community and land. This dynamic understanding also recognizes that food sovereignty is embedded in broader systems—including the legacies of colonialism, the assertion of Indigenous rights, and the struggle for self-governance—highlighting its role in both societal transformation and cultural continuity [31,32]. Therefore, it must remain responsive to each community’s unique environment, history, and social structure [31,33]. Colonial policies and practices have historically disrupted Indigenous food systems, displacing communities from ancestral lands, restricting access to traditional food sources, and imposing agricultural systems that conflict with Indigenous knowledge. These disruptions have significantly eroded Indigenous health, cultural continuity, and ecological wisdom. In response, IFS seeks to restore not only access to food but also the cultural, spiritual, and ecological relationships that underpin Indigenous food systems. In this context, a critical distinction emerges between IFS and broader food sovereignty frameworks. Although both emphasize local control, justice, and resistance to industrialized food systems, IFS is rooted in Indigenous ontologies, cultural responsibilities, and relationships with land and creation that predate and transcend colonial paradigms [11,18,30,34]. It is not merely an adaptation of food sovereignty; rather, it is a reclamation of what is inherently Indigenous—ways of living and relating to food that are deeply tied to identity, governance, and spiritual responsibilities [35]. Although broader food sovereignty movements often focus on rights-based approaches and resistance to corporate control, IFS expands these principles by centering the value of food in holistic well-being—physical, social, spiritual, emotional, and intellectual—and fostering accountability to land, food systems, and community relationships [11,36,37]. It also reflects the broader context of Indigenous peoples’ rights and the ongoing impacts of colonization, situating food not only as nourishment but also as central to cultural resurgence, sovereignty, and societal transformation. For Indigenous communities, reclaiming food sovereignty is inherently tied to self-governance, cultural restoration, and land stewardship, including access to sacred spaces for ceremony and traditional practices [38]. IFS also emphasizes cultural identity and relationality, framing food as a sacred element that connects individuals, communities, and the land. Coté [11] and Hauptmann [14] illustrate how IFS embodies kinship-based responsibilities extending to all living beings, reinforcing the idea that food sovereignty is grounded in relational values that promote social and environmental responsibility.

In summary, IFS sets itself apart from the broader food sovereignty movement by asserting ontological principles of relationality, cultural continuity, and spiritual connections to the land. Although both movements share some foundational concerns, IFS is a useful and powerful tool outlining essential components, and it reclaims what is inherently and rightfully Indigenous—rooted in longstanding struggles against colonial dispossession and grounded in cultural responsibilities. It is both a process and a goal: a pursuit of self-determined food systems and a state of sovereignty that communities continually define and enact based on their ecological and cultural contexts. As the reviewed studies show, IFS is more than a political framework; it is a culturally aligned and ecologically responsible approach that affirms the rights of Indigenous communities to define, sustain, and adapt their food systems in alignment with their values and traditions, making it a flexible yet powerful means of reclaiming and maintaining control over food systems.

## Section 2: Principles of IFS

IFS is deeply rooted in cultural, spiritual, and ecological values that contrast sharply with the Eurocentric, extractive models dominating global food systems. The studies reviewed reveal several recurring principles that underpin IFS, capturing the holistic and relational nature of food sovereignty within Indigenous communities (Supplemental Table 1). These principles reflect recurring concepts identified across the included studies and grouped into broader thematic categories. The main principles include the following: *1*) sacred relationships with food and land; *2*) self-determination and decision-making power; *3*) active participation in food systems; *4*) community involvement and cultural restoration; *5*) inclusion and promotion of Indigenous knowledge; *6*) policy and legislative advocacy; *7*) environmental stewardship and sustainability; *8*) relationality, responsibility, and reciprocity; and *9*) access to resources and control over food systems. Although presented as distinct themes, they are often interconnected in practice, reflecting the relational and holistic nature of IFS.

A central theme across the literature is the “sacred relationship with food and land.” Food is not merely a resource but a sacred gift, imbued with cultural and spiritual significance [9,12]. This principle, which Morrison [9] describes as “sacred or divine sovereignty,” emphasizes that food is a creation of the land and the Creator, and it must be treated with respect and reciprocity. Martens et al. [39] further emphasize this principle in practice, noting that IFS is a “living reality” grounded in interdependent relationships with the land, and that food must be treated as sacred. The Nuu-chah-nulth Nation, for instance, upholds the principle of *hishuk’ish tsawalk* or “everything is one,” underscoring interconnectedness and the sacred duty to care for all life [11,40]. This relationship guides practices that honor the earth, reinforcing that food systems must align with ecological balance and cultural obligations.

“Self-determination and decision-making power” are consistent core principles in IFS. In this context, Indigenous communities should independently manage and control their food systems according to cultural values, with freedom in decision-making related to food production, resource access, and land use [19,35,[41], [42], [43]]. Self-determination, as Morrison [9] and subsequent studies [16,23,44] highlight, opposes the colonial structures that historically undermined these rights. Examples include communities establishing local governance systems, resisting imposed agricultural policies, and revitalizing traditional foodways [17,[44], [45], [46]].

“Active participation in food systems,” through practices such as hunting, fishing, gathering, gardening, and sharing, is a cornerstone of IFS, emphasizing autonomous food production and traditional practices [24]. Participation is not merely a means of sustenance; it is a way of preserving cultural practices, strengthening community bonds, and fostering collective resilience. Cidro et al. [47] illustrate how this principle can begin in infancy, highlighting breastfeeding and the early introduction of country foods as foundational expressions of food sovereignty that nourish both body and spirit. In other contexts, studies describe urban Indigenous communities using community gardens and food-sharing initiatives to reinforce cultural identity and social responsibility [25,41,48]. These activities underscore the principle that sovereignty is practiced actively through daily involvement and intergenerational knowledge sharing [22,49,50]. Robin [21] similarly describes IFS as a “living, breathing way of life” grounded in history, identity, and relationships to land, sustained through daily practices.

“Community involvement and cultural restoration” are essential to IFS. Many studies highlight that IFS is realized through communal participation in decision-making, knowledge-sharing, and food-related practices. Through community activities, Indigenous peoples sustain cultural knowledge and foster resilience against socioeconomic and environmental challenges. Community-driven initiatives such as food-sharing programs, educational workshops, efforts to revitalize Indigenous languages, and land-based youth farming programs serve as sites of cultural restoration, passing on traditional knowledge, beliefs, and practices to younger generations [18,19,22,42,51,52].

“Inclusion and promotion of Indigenous knowledge” is another recurring theme, with IFS often defined by the integration of Indigenous ecological and agricultural knowledge into food practices. This includes techniques such as seasonal hunting and sustainable harvesting that align with the cycles of the natural world, thereby preserving biodiversity and ensuring food security [53]. Traditional ecological knowledge (TEK) is foundational in guiding environmentally sustainable food practices and ensuring that these practices are contextually and culturally relevant [53,54]. Some communities have revitalized seasonal calendars and indicators, such as animal behaviors and moon cycles, to guide planting and harvesting practices in accordance with local ecological knowledge [53].

“Policy and legislative advocacy” plays a critical role in supporting IFS. To fully realize food sovereignty, Indigenous communities and advocates call for policies that protect Indigenous rights to land and access to traditional resources. Legislative advocacy, as Morrison [9] articulates, is essential for addressing the colonial frameworks that often restrict these rights. Kamal et al. [26] and others argue for legal reforms that recognize and enable Indigenous communities to operate sustainable food systems autonomously, securing access to vital resources such as land and water [28].

“Environmental stewardship and sustainability” are deeply embedded in IFS principles, emphasizing the necessity of protecting natural resources. Indigenous communities view food sovereignty as inseparable from the health of ecosystems, which includes responsible stewardship of land, water, and biodiversity [15,31]. Sustainable practices informed by TEK ensure that natural resources are managed in a way that respects ecological limits and supports future generations. Blue Bird Jernigan et al. [33] and Blanchet et al. [55] provide examples where Indigenous communities use TEK-based approaches to harvest resources sustainably, reinforcing environmental stewardship as a central value.

“Relationality, responsibility, and reciprocity” are fundamental to IFS, emphasizing mutual respect and interconnectedness between humans, plants, animals, and the environment [18,26,27,34,35,37,40,41,51,[56], [57], [58]]. IFS practices are structured around kinship ties and community responsibilities that extend beyond human interests to include the welfare of nonhuman beings and the land [11,46,[59], [60], [61], [62]]. This holistic view is rooted in a belief that food is sustained through relationships of care and respect, which contribute to resilience and social cohesion. Reciprocity is not limited to food gathering and production but encompasses the social relationships that food sustains, creating a holistic view of sovereignty that connects food with broader social and environmental responsibilities, a perspective central to studies by Brant et al. [18], Miltenburg et al. [51], Stevens et al. [63], and others [32,59,64].

“Access to resources and control over food systems” is another principle commonly cited in the literature. Studies repeatedly affirm that food sovereignty includes the right to manage and control one’s own food sources, along with fair access to land and water. This control over resources is often linked to the broader decolonization movement, where Indigenous communities reclaim access to traditional territories and food systems disrupted by colonial interventions [15,33,[64], [65], [66]].

Together, these principles reflect IFS as a paradigm that encompasses self-determined, culturally aligned food systems. The reviewed studies underscore that IFS is more than a political framework; it is a comprehensive and ethical approach to food, rooted in relational values and ecological responsibilities that guide both everyday practices and long-term strategies for resilience.

### Expanding indigenous food sovereignty: community initiatives and global recognition

The IFS movement has grown in response to the need to address ongoing challenges faced by Indigenous communities, such as environmental degradation, climate change, and socioeconomic marginalization. Indigenous communities across Canada, the United States, and other regions have adapted the principles of food sovereignty to their specific cultural and ecological contexts. In British Columbia, for instance, the Working Group on IFS was formed to promote Indigenous perspectives within the broader food sovereignty movement and advocate for culturally relevant food policies [9]. This group, along with other Indigenous-led initiatives, has been instrumental in raising awareness about the unique aspects of IFS and advocating for systemic changes that respect Indigenous rights and knowledge systems.

The UNDRIP and the UNDROP have also contributed to the growing recognition of IFS as a fundamental human right [5,6]. Both declarations affirm the rights of Indigenous peoples and rural communities to control their food systems and uphold traditional food practices. UNDRIP, in particular, supports Indigenous peoples’ rights to self-determination, land, and resources, laying a foundation for the formal recognition of IFS within international law [5]. UNDROP similarly emphasizes the right to culturally appropriate food and self-determined agricultural systems, aligning with the principles of IFS by acknowledging the importance of local knowledge and community governance in sustainable food systems [6].

The evolution of food sovereignty as a global movement brought attention to issues of local control, food access, and resistance to industrial food systems. Within this context, the concept of IFS gained visibility, highlighting principles that Indigenous peoples have long practiced through their relationships with the land, food systems, and cultural responsibilities. Although linked to the broader food sovereignty discourse, IFS reflects deeply rooted worldviews that prioritize Indigenous rights, self-determination, land stewardship, and intergenerational knowledge. It is a dynamic and adaptive framework that not only addresses food security but also seeks to reclaim and revitalize the cultural, spiritual, and ecological dimensions of Indigenous food systems. As such, IFS is not only a pathway toward decolonization and the restoration of Indigenous identities, health, and resilience, but it is also an active movement, a journey, and a struggle rooted in self-determined, sustainable food practices.

## Section 3: Tools for Evaluating IFS

Assessing IFS is a multifaceted task requiring tools and methodologies that are sensitive to the cultural, spiritual, and ecological values central to Indigenous communities’ food systems. Recognizing the holistic and community-centered nature of IFS, a variety of assessment tools and frameworks have been adapted or developed, with an emphasis on Indigenous methodologies, participatory processes, and culturally relevant and specific metrics.

One prominent approach to evaluating IFS is the Food Sovereignty Assessment Tool (FSAT), developed by the First Nations Development Institute. FSAT offers a structured method for assessing community food sovereignty by examining aspects such as access to traditional foods, control over food sources, and the economic impact of food systems within Indigenous communities. This tool operates through a participatory process, engaging community members in identifying local food assets, needs, and priorities, thus ensuring that the assessment aligns closely with community perspectives and aspirations [33,67].

Building on FSAT’s foundation, researchers have highlighted core principles essential to evaluating IFS, including community ownership, traditional food knowledge inclusion, promotion of cultural foods, and environmental sustainability. In a systematic review, Abdul et al. [67] noted that assessment approaches incorporating these principles more accurately reflect the unique values and priorities of Indigenous communities. Community ownership, for instance, is often evaluated based on the involvement of Indigenous members in the planning, implementation, and sustainability of food projects, whereas environmental sustainability considers the ecological stewardship that Indigenous communities emphasize within their food systems [54].

Recent research also emphasizes the need for adaptable indicators to evaluate IFS across diverse contexts. For example, Binimelis et al. [65] proposed adapting existing food sovereignty indicators to better fit Indigenous frameworks, thereby aligning them with the cultural and ecological dimensions unique to each community. These indicators include measures for resource access, food production, and the sustainability of practices, all tailored to the relational and cultural responsibilities emphasized in IFS [65].

Furthermore, Blue Bird Jernigan et al. [33] developed a comprehensive set of food sovereignty indicators specifically designed to support Indigenous capacity building and health. These indicators span 7 domains: access to resources, production, trade, food consumption, policy, community involvement, and cultural relevance. This adaptable framework allows for evaluation in local contexts across various Indigenous communities, helping communities gauge their progress in developing sustainable and autonomous food systems that align with cultural practices [33]. The authors emphasize that food sovereignty efforts depend on Indigenous self-governance, influence over policy, and community-based decision-making structures such as food councils, which are essential to maintaining control over food systems and ensuring long-term sustainability [33].

CBPR is another critical approach used to integrate Indigenous perspectives throughout the research process. CBPR aims to shift power dynamics by fostering collaborative partnerships in which Indigenous communities lead or co-lead the research direction and methodologies, ensuring that their perspectives and priorities shape the outcomes. Many IFS assessments using CBPR also incorporate protocols around data sovereignty, ensuring that data generated from these assessments stay under the control and benefit of Indigenous communities [67].

### Challenges of existing tools in diverse settings

Despite the value of these tools, applying them across different Indigenous communities and ecological settings poses several challenges:

1) Adaptability to diverse ecologies*:* Existing tools are sometimes difficult to adapt to the varied ecological landscapes inhabited by Indigenous communities [59]. Standard indicators may not account for region-specific resources, climate conditions, or seasonal patterns, which are vital to accurately capturing Indigenous food practices. For instance, Binimelis et al. [65] proposed adapting general food sovereignty indicators to better align with Indigenous frameworks, emphasizing localized measures for resource access, food production, and sustainability that match each community’s ecological and cultural context.
2) Community and cultural relevance: Tools developed with standardized indicators may lack cultural relevance for Indigenous communities with unique traditions and values. FSAT, e.g., provides a structured framework but may need community-specific adaptations to capture the depth of each culture’s food sovereignty goals. The flexibility to customize these tools based on community feedback is essential for maintaining cultural relevance in assessments [33,67].
3) Resource constraints: Implementing IFS assessment tools in remote or resource-limited areas can be challenging due to constraints in infrastructure, technical support, and financial resources. Although CBPR is valuable for its participatory focus, its successful application often depends on sustained engagement and funding, which may not be feasible for every community, particularly those facing socioeconomic barriers.
4) Changing traditional food access: Climate change, habitat loss, and environmental degradation impact traditional food sources, making it difficult for static indicators to capture these dynamic conditions. This challenge highlights the need for adaptable indicators that can accurately reflect shifting access to and sustainability of traditional foods, considering the increasing pressures on Indigenous food systems [33,65].

### Evaluating decolonizing efforts supported by these tools

A critical component of IFS assessment is determining whether these tools contribute to decolonizing food systems by centering Indigenous perspectives, promoting self-determination, and addressing historical colonial impacts on Indigenous foodways.

1) Community ownership and data sovereignty: Tools that support decolonization prioritize community ownership of data, ensuring that information remains under Indigenous control. CBPR excels in this regard by embedding community members in every stage of the assessment, from design to analysis, and reinforcing data sovereignty, where communities retain control over how their data are used [67].
2) Integration of Indigenous knowledge and practices: Effective IFS tools should incorporate Indigenous knowledge systems, traditional practices, and ecological stewardship, countering Eurocentric, extractive models. For example, Blue Bird Jernigan et al. [33] developed food sovereignty indicators that are tailored to Indigenous ecological knowledge, encompassing traditional practices in sustainable land use, food production, and community food sharing.
3) Empowering policy influence: A key element of decolonization is enabling Indigenous communities to use assessment outcomes to advocate for policy changes that recognize and support their food sovereignty. Effective tools should equip communities with data to demonstrate the benefits of food sovereignty for health, cultural continuity, and environmental stewardship, strengthening their case for policy reform that respects Indigenous autonomy [54,65].
4) Sustaining Indigenous-led adaptations: Although tools such as FSAT and CBPR frameworks support decolonizing objectives, their effectiveness depends on the continued engagement of Indigenous leadership and the resources needed for long-term adaptations. Without sustained support, Indigenous communities may struggle to maintain the adaptations that make these tools effective for decolonizing purposes, emphasizing the need for ongoing capacity building and funding [33].

In summary, the tools and methodologies used to assess IFS reflect the values, traditions, and resilience strategies embedded in Indigenous peoples’ food systems. By emphasizing Indigenous-led research, community participation, and culturally specific indicators, these tools not only measure food sovereignty outcomes but also advance broader decolonizing efforts. However, challenges such as ecological adaptability, cultural relevance, and resource constraints highlight the need for further refinements and sustained support. Although these tools capture several important dimensions of IFS, they do not consistently reflect the full range of principles identified in this review across diverse contexts. In addition, although this review focused on structured tools and indicators, we did not identify studies that explicitly describe Indigenous methodologies as formalized evaluation tools. This suggests an opportunity for future research to further develop Indigenous-led approaches to evaluation. More broadly, these limitations reflect the inherent difficulty of assessing relational, cultural, and governance dimensions through standardized approaches, because these dimensions are context-specific, dynamic, and embedded in lived experiences and relationships that are not easily captured through fixed indicators. Effective IFS assessment tools, therefore, provide Indigenous communities with a means of asserting their food sovereignty, advocating for policy change, and fostering resilience through culturally aligned and environmentally sustainable practices.

## Section 4: Practical Examples and Daily Life Practices in IFS

IFS is not only theorized in academic literature but actively enacted by Indigenous communities through intentional, community-led initiatives that revitalize traditional food practices, enhance resilience, and foster cultural continuity. The examples presented here reflect communities and Nations that have explicitly framed their work as part of food sovereignty efforts or whose initiatives are grounded in principles aligned with IFS as defined by those communities. These examples provide valuable insights into the successes and everyday practices that reinforce IFS constructs, highlighting how Indigenous peoples adapt and assert food sovereignty in the face of contemporary challenges. They illustrate that IFS is not an abstract concept imposed by researchers but a lived, evolving movement rooted in community-defined goals, responsibilities, and governance. These examples also illustrate how the core principles of IFS identified in Section 2 are enacted in practice across diverse contexts, providing concrete expressions of concepts such as self-determination, relationality, environmental stewardship, and the transmission of Indigenous knowledge.

### Sacred relationships with food and land

Many examples in the literature emphasize the deep cultural and spiritual relationships that Indigenous communities maintain with their lands. The Nuu-chah-nulth Nation, e.g., embodies the principle of *hishuk’ish tsawalk* (“everything is one”), illustrating how their food sovereignty efforts, such as communal fishing and gardening projects, reinforce this worldview [11]. This spiritual respect for land and food sources is echoed across other communities, such as the Inuit in Kalaallit Nunaat (Greenland), who value *kalaalimerngit* (traditional foods) as a cultural foundation and as part of broader efforts to uphold self-determined food practices [14].

### Self-determination and Indigenous governance

IFS often involves reclaiming decision-making powers and governance structures. For example, the Oneida Nation of Wisconsin’s food sovereignty initiatives, such as corn cultivation, underscore the importance of local control over food production and distribution [68]. This focus on self-determination is also seen in the Fisher River Cree Nation’s programs in Canada, which engage youth in traditional food practices as part of a broader, community-driven vision of food sovereignty and cultural renewal, while reinforcing local governance over food systems [19]. Similarly, the Anishinaabe Nation of Biigtigong Nishnaabeg practices autonomous wildlife monitoring and holds annual moose hunt camps, where families gather to harvest and process moose while transmitting knowledge across generations—an approach that supports both food sovereignty and Indigenous-led governance [45]. The Nuu-chah-nulth Nation also engaged in a decade-long legal battle affirming their right to implement culturally grounded fishing and harvesting strategies, reinforcing the importance of policy reform and cultural rights in IFS [23]. In the United States Midwest, Ojibwe communities continue to protect and harvest wild rice, asserting treaty rights and resisting commercial and genetic interventions in defense of their cultural and spiritual responsibilities to the plant and its ecosystem [57].

### Cultural restoration and transmission of Indigenous knowledge

The emphasis on teaching traditional food practices to younger generations is a recurring theme. Numerous initiatives across Indigenous communities—including O-Pipon-Na-Piwin Cree Nation—demonstrate how intergenerational knowledge-sharing, community gardening, and land-based education serve as powerful pathways for cultural revitalization and food sovereignty [12,13,15,16,19,39,47,51,64]. Programs such as the Outdoor Education Program at Fisher River Cree Nation facilitate knowledge-sharing across generations by incorporating traditional hunting, fishing, and gathering into high school curricula, linking education to cultural continuity [19]. In urban areas, the Northern Arapaho’s Growing Resilience Project uses community gardening as a strategy for healing and cultural revitalization, engaging youth and elders alike to strengthen cultural identity and pass down traditional knowledge [69]. Similarly, the Youth Leadership Training Program at MA‘O Organic Farms in Wai‘anae, Hawai‘i supports Native Hawaiian youth in organic farming, health education, and cultural reconnection, reinforcing food sovereignty through land-based learning and community food contributions [52]. In Kalapuya ilihi (Oregon), the revitalization of Three Sisters agriculture through educational fieldwork and community-university collaboration fosters youth learning and cultural reconnection, advancing food sovereignty through the restoration of Indigenous ecological knowledge and relationships to land [70].

Across different regions, Indigenous communities also integrate spiritual and ceremonial practices into their food sovereignty efforts, honoring food as a sacred relationship that embodies cultural identity and environmental stewardship. Programs focused on youth engagement and intergenerational learning are particularly important for transferring cultural knowledge and ensuring the continuity of traditional foodways [26,36,42]. In Mi’kmaq territory, the Seven Paddles Project engaged youth in retracing ancestral canoe routes as a way of fostering cultural identity, food gathering skills, and political consciousness grounded in treaty rights [28]. Similarly, in First Nations communities across Canada, Learning Circles have engaged elders and youth in seasonal food harvesting, school-based lunch programs, and traditional food preparation to strengthen local food systems and reinforce cultural transmission [71]. Through activities such as communal fishing, gardening, seasonal ceremonies, and structured educational on-the-land learning programs, these initiatives foster self-determination, resilience, and sustainable relationships with the land, while strengthening bonds and promoting cultural renewal [27,58,62,72].

### Environmental stewardship and sustainability

Sustainability and environmental stewardship are central to many IFS initiatives. For instance, Māori communities in Aotearoa rely on *maramataka* (lunar calendar) to guide their planting and harvesting, ensuring alignment with environmental cycles as a part of self-determined food systems rooted in Māori cosmology and sovereignty [60,61]. Similar practices are observed among Quechua communities in Peru, where rituals honoring Pachamama (Mother Earth) are integrated into sustainable farming practices that reflect Indigenous values of autonomy, balance, and responsibility within food systems [60,61]. Nuu-chah-nulth communities on Vancouver Island demonstrate IFS through marine stewardship rooted in traditional governance. Guided by principles such as *hišukʔiš c̓awaak* (“everything is one”), they manage sea otter populations to protect shellfish stocks, maintain ecological balance, and uphold reciprocal relationships with the environment [40]. In the Great Lakes region, Anishinaabe communities have led wild rice restoration efforts as a form of ecological stewardship and resistance to settler-colonial degradation, reaffirming the cultural and ecological centrality of wild rice within Indigenous food systems [31,32].

### Active participation in food systems

Numerous communities implement community gardens to foster local food production and enhance cultural education within food sovereignty frameworks defined by the communities themselves [19,24]. For instance, urban Indigenous communities in the Grand River Territory use community gardening not simply as a food access strategy, but as a deliberate means to reclaim cultural teachings, food practices, and land-based relationships in alignment with IFS principles [51]. Such gardens are not only sources of sustenance but also serve as cultural hubs where Indigenous foods, values, and agricultural knowledge are shared [16,27]. Practices such as harvest sharing, community gardening, and ceremonial food preparation are widely recognized as integral expressions of IFS, reinforcing collective responsibility, interconnection, and cultural resurgence [18].

### Relationality, reciprocity, and cultural practices

Indigenous communities frequently incorporate ceremonial practices into their food sovereignty efforts to honor cultural identity and communal ties. These initiatives include wild-food banks distributing moose meat, traditional medicine camps, community hunts, and land-based healing programs rooted in cultural resurgence and community well-being [56]. The Oneida Nation’s husking bee festival celebrates the harvest of corn, symbolizing resilience and shared cultural heritage, and is framed as part of a broader community effort to revitalize Haudenosaunee food systems and governance [63]. Similarly, the Syilx Okanagan’s salmon restoration initiative includes traditional feasts and ceremonies that not only reinforce their cultural and spiritual connection to salmon as a vital food source but also reflect broader efforts to restore ecosystems essential to Indigenous food systems and sovereignty [55,73]. In the urban context of California, the Sogorea Te’ Land Trust illustrates how land rematriation supports IFS by creating sacred space for ceremony, restoring traditional foodways, and reasserting Indigenous land governance through daily practices such as planting, ceremony, and collaboration with food justice organizations [38]. In several Eastern United States communities, traditional growing practices, seed preservation, and youth land-based education also reflect IFS in action [15].

These examples illustrate the multifaceted role of IFS initiatives in reinforcing cultural values, promoting sustainable practices, and strengthening community resilience. By adapting food sovereignty principles to unique ecological and cultural contexts, Indigenous communities showcase the diversity and adaptability of these efforts. Central to these initiatives is the deliberate and self-defined enactment of IFS—through practices that reflect longstanding responsibilities to land, food, and culture. These actions reinforce Indigenous identity, self-determination, and resilience, achieved through traditional practices, communal activities, and agroecological methods that support both cultural renewal and environmental sustainability [25,51,74]. These examples were drawn from studies that met the inclusion criteria and reflect explicit efforts by Indigenous communities to reclaim control over their food systems in accordance with their own values, laws, and governance. This intentionality distinguishes them from general food programming and reflects the political, cultural, and ecological dimensions of IFS as described throughout this review. Together, these examples underscore a shared commitment to reclaiming food systems that reflect Indigenous values, build community cohesion, and create resilient food networks that honor heritage and promote ecological balance. In some cases, these examples provide more detailed and context-specific expressions of the principles identified in the literature, highlighting how IFS is lived and practiced within specific communities and reinforcing the importance of examining real-world contexts alongside conceptual frameworks. In doing so, these examples often reflect multiple, overlapping principles of IFS, illustrating how its dimensions are interconnected and expressed simultaneously in practice.

### Strengths and limitations

This review’s methodological rigor lies in its structured and comprehensive search and screening processes, adhering to PRISMA-ScR guidelines for scoping reviews, which help ensure transparency and replicability. A key strength is the focused exploration of IFS, allowing for an in-depth analysis of the concept as it pertains to self-determined food systems. By concentrating specifically on IFS rather than the broader scope of food sovereignty or Indigenous food security, this review delves deeply into the cultural, ecological, and social dimensions central to Indigenous perspectives, with a particular emphasis on Indigenous authors’ viewpoints to support a decolonized approach to the overall concept.

Despite these strengths, several limitations should be considered. The search strategy, although intentionally focused, may not have captured all relevant studies because of the varied terminology used to describe Indigenous populations and communities. The use of a limited set of search terms reflects a deliberate methodological choice aligned with the objectives of this review, which focused on identifying and synthesizing definitions and key principles explicitly related to “IFS.” Although this approach ensured conceptual specificity, it may have excluded studies addressing similar concepts using different terminology.

Articles that referenced specific community names (e.g., Amazonian food sovereignty; Inuit food sovereignty) were included during screening to partially mitigate this limitation [24,59]. However, given the diversity and number of Indigenous communities globally, it was not feasible to systematically include all possible community-specific search terms. As such, some relevant studies using community-specific terminology rather than the broader term “Indigenous food sovereignty” may not have been captured.

Another limitation relates to language restrictions; studies were eligible for inclusion if published in English or French (the languages spoken by the first author), which may have led to the exclusion of pertinent studies published in other languages. In addition, the review focused solely on the term “Indigenous food sovereignty” in the search terms, without incorporating broader synonyms or related terms. Although this limitation was mitigated by including studies that referenced community-specific food sovereignty, it is possible that studies discussing IFS under other terms were not captured. Because the term “Indigenous food sovereignty” emerged later, some studies involving South American communities may have discussed IFS but used the more general term “food sovereignty,” as introduced by La Via Campesina.

Furthermore, publication bias may have influenced the results, as studies not explicitly focused on IFS in their titles or abstracts could have been unintentionally excluded. The review primarily included peer-reviewed articles but also some gray literature in the form of reports identified through Google Scholar. However, as gray literature was not systematically searched, and other forms such as theses, books, and websites were not included, the scope of the review may be limited, potentially omitting valuable insights and practical examples of IFS that are not represented in peer-reviewed literature. In addition, many Indigenous communities do not prioritize academic publishing unless in collaboration with researchers, as their focus often lies in community-based action and knowledge transmission rather than dissemination through academic channels. As a result, the literature included here likely represents only a portion of the broader efforts and knowledge related to IFS. Future research would benefit from more systematically incorporating gray literature and community-based sources to better reflect Indigenous knowledge systems and the lived experiences of IFS.

In conclusion, this review has examined IFS as a concept grounded in the peer-reviewed literature, with particular attention to perspectives articulated by Indigenous scholars and communities. Through the studies reviewed, IFS emerges as a holistic framework described by key principles—such as the sacred relationship with food and land; self-determination and decision-making power; active participation in food systems; community involvement and cultural restoration; the inclusion and promotion of Indigenous knowledge; policy and legislative advocacy; environmental stewardship and sustainability; relationality, responsibility, and reciprocity; and access to resources and control over food systems. These principles, as articulated in the literature, emphasize that IFS is about more than food access; it is a means of upholding cultural identity, environmental stewardship, and self-determination.

Drawing on examples from the literature and Indigenous perspectives, this review highlights how Indigenous communities are reclaiming food sovereignty through daily practices, sustainable resource management, and adaptation to contemporary challenges. These efforts contribute not only to Indigenous food security but also to broader goals of cultural preservation, environmental sustainability, and community resilience, with direct implications for improving health and well-being through land-based and culturally grounded food practices. By focusing on Indigenous-led approaches, this article underscores the importance of respecting and protecting Indigenous knowledge and practices within food sovereignty efforts.

To fully support IFS, governments and organizations must recognize the inherent rights of Indigenous peoples to control their food systems. This includes restoring land rights, safeguarding traditional knowledge, and ensuring resources for sustainable food production aligned with Indigenous values. Such recognition, in line with international frameworks such as the UNDRIP and the UNDROP, is essential for Indigenous communities to sustain and strengthen their food sovereignty.

In conclusion, IFS represents a pathway toward decolonization, cultural renewal, and resilience in the face of external pressures. By supporting Indigenous-led approaches and understanding food sovereignty through Indigenous perspectives, this review contributes to a more inclusive and culturally grounded understanding of food systems. Future research should focus on developing and refining culturally grounded and Indigenous-led approaches and tools to assess IFS across diverse contexts, ensuring that these approaches are responsive to the range of principles identified in this review, while remaining adaptable to local cultural, ecological, and governance realities. It should also examine how these principles are operationalized across different ecological, cultural, and governance settings and their implications for health and well-being. Such efforts, alongside continued policy advocacy, are needed to support IFS and ensure more sustainable and equitable food systems for future generations.

## Author contributions

The author’s responsibilities were as follows – IS: conceptualized and designed the study, conducted the literature search, screening, and data extraction, and drafted the manuscript; GBC: contributed to screening and data extraction; CF, AA, TD: contributed to conceptualization, interpretation, and critical revisions; and all authors: read and approved the final manuscript.

## Data availability

Data described in the manuscript, including extracted data from included studies, are available within the article and its supplementary materials. Supplemental Material related to the screening and selection process is available from the corresponding author upon reasonable request.

## Funding

This review was conducted as part of the Ărramăt Project, which is funded by the New Frontiers in Research Fund and Asociación ANDES, and supported by the Centre for Indigenous Peoples’ Nutrition and the Environment. We acknowledge the support of the Government of Canada’s New Frontiers in Research Fund (NFRF), [NFRFT-2020-00188].

**Figure:**
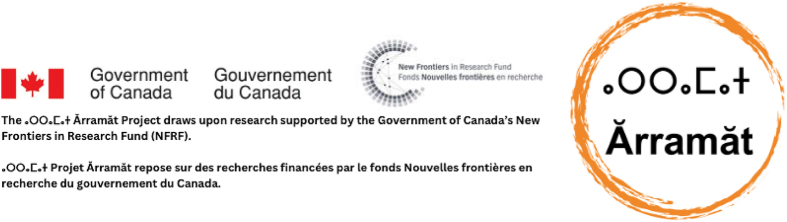

## Declaration of generative AI and AI-assisted technologies in the writing process

The authors declare that no generative AI or AI-assisted technologies were used in the writing of this manuscript.

## Conflict of interest

TD is supported by the Canada Research Chair Program. All other authors report no conflicts of interest.
