# Supplementary material for: Decolonizing the Narrative of Indigenous Food Sovereignty: A Scoping Review
Source: Curr Dev Nutr. 2026 Jul 2;10(8):109430. doi: 10.1016/j.cdnut.2026.109430 (PMC13475507; doi:10.1016/j.cdnut.2026.109430)
Supplement: Supplementary file 1 [file mmc1.docx]

**Supplementary Table 1:** Study Selection for the Scoping Review on Indigenous Food Sovereignty

| **Author**  **(year)** | **Study design** | **Community and Region** | **Definition of Indigenous Food Sovereignty & Food Security** | **Key Elements of Food Sovereignty & Security** | **Tools to Assess Food Sovereignty** | **Case Studies & Examples from Daily Life Practices** |
| --- | --- | --- | --- | --- | --- | --- |
| **“Indigenous food sovereignty assessment—A systematic literature review”** [**https://doi.org/10.1111/1747-0080.12813**](https://doi.org/10.1111/1747-0080.12813) | | | | | | |
| Abdul et al.  (2023)^(1)^  (Two Australian Indigenous  Researchers and five members of non-Australian Indigenous  Backgrounds) | Systematic literature review | Indigenous communities worldwide, including examples from Canada, the U.S., and other regions | Indigenous food sovereignty is described as “a rights-based approach to land, food, and the ability to control a production system that emphasizes accountability to holding culturally, ecologically, and spiritually respectful relations with plants, animals, environment, and surrounding communities within those systems”. *Maudrie et al. 2021* | The four key domains of Indigenous food sovereignty *Maudrie et al. (2021)*:  1. Community ownership  2. Inclusion of traditional  food knowledge  3. Inclusion and promotion of cultural foods  4.Environmental or intervention sustainability. | The Food Sovereignty Assessment Tool, developed by First Nations Development Institute, in the USA. *Used in intervention projects, it has not been used in research literature.*  The Traditional Food Frequency Questionnaire and Household Food Security Survey Model were commonly adapted to assess traditional and local food inclusion. | - |
| **“Elements of Successful Food Sovereignty Interventions within Indigenous Communities in the United States and Canada: a Systematic Review”** [**https://doi.org/10.1016/j.cdnut.2023.101973**](https://doi.org/10.1016/j.cdnut.2023.101973) | | | | | | |
| Gutierrez et al.  (2023)^(2)^  (Indigenous author) | Systematic review of food sovereignty interventions | Indigenous communities across the U.S. and Canada | Indigenous food sovereignty (IFS) has been determined to be a mechanism for Indigenous communities to build their capacity to address food insecurity.  Food sovereignty “the right of peoples to healthy and culturally appropriate food produced through ecologically sound and sustainable methods, and their right to define their own food and agriculture systems” *Maudrieet al. (2021)* | The study identifies four main elements essential to successful IFS interventions:  -Transmission of Knowledge and Skills: workshops often led by Elders.  -Cultural Connectedness through Cultivation Practices: Gardening and cultivation practices that strengthen cultural identity and community bonds.  -Preparation and Consumption of Traditional Foods: Community programs supporting access to and consumption of traditional foods.  -Community-based Partnerships and Collaborations: Partnerships with organizations, universities, and community stakeholders to bolster food sovereignty efforts. | - | Examples include the use of community gardens, food-sharing initiatives, and educational workshops that promote traditional food practices and knowledge transmission. Specific interventions mentioned include gardening programs and traditional food programs like the community freezer initiative in Nunatsiavut, which helps preserve wild foods for future use. |
| **“A Scoping Review of the Use of Indigenous Food Sovereignty Principles for Intervention and Future Directions”** [**https://doi.org/10.1093/cdn/nzab093**](https://doi.org/10.1093/cdn/nzab093) | | | | | | |
| Maudrie et al.  (2021)^(3)^  (Indigenous author) | Scoping Review | Indigenous communities in North America, U.S. and Canada | Indigenous food sovereignty can be defined as a rights-based approach to land, food, and the ability to control a production system that emphasizes accountability to holding culturally, ecologically, and spiritually respectful relations (with plants, animals, environment, and surrounding communities) within those systems.  Food sovereignty was defined by the first global Forum for Food Sovereignty as “the right of peoples to healthy, culturally appropriate food produced through ecologically sound and sustainable methods, and their right to define their own food and agriculture systems. | The review identifies four main principles of Indigenous food sovereignty:  -Community Ownership: Interventions involve community-led planning, implementation, and sustainability efforts.  -Inclusion of Cultural Food Knowledge: Programs include traditional knowledge sharing, such as storytelling and intergenerational teaching.  -Promotion of Traditional Foods: Encourages the inclusion of culturally significant foods in diets.  -Environmental Sustainability: Programs support sustainable, ecologically responsible practices and long-term environmental impacts. | The study developed a scoring system to evaluate the application of food sovereignty principles in interventions. IFS Scores: Intended to provide an indication of the degree to which an intervention is providing supporting for food sovereignty in the community (or communities) they are working with. | - Creation or use of community research councils (or community advisory boards): community ownership, was demonstrated with a CBPR approach and specific mentions of how the intervention was guided or led by community.  Berry picking, gardening, harvesting, and cooking lessons: cultural food knowledge, was supported through intervention activities that were tribally or regionally specific  -Fish to School Program: A program providing traditional fish in schools, increasing access to traditional foods.  -Community Gardens and Food Store Programs: These programs increase the availability of traditional foods and support sustainable practices, reinforcing cultural connections. |
| **“A holistic definition of healthy traditional harvest practices for rural Indigenous communities in Interior Alaska”** [**https://doi.org/10.5304/jafscd.2019.09B.009**](https://doi.org/10.5304/jafscd.2019.09B.009) | | | | | | |
| Heeringa et al. (2019)^(4)^  (Include Indigenous authors) | Case Study | Athabascan Indigenous Communities Interior Alaska | Inuit Circumpolar Council describe Inuit food sovereignty as: The right of Alaskan Inuit to define their own hunting, gathering, fishing, land, and water policies, the right to define what is sustainably, socially, economically, and culturally appropriate for the distribution of food and to maintain ecological health; the right to obtain and maintain practices that ensure access to tools needed to obtain, process, store, and consume traditional foods. | Key elements include ecological health, decision-making power, cultural identity, sharing, interdependence, and community resilience.  Food sovereignty by definition carries inherent meanings of autonomy, authority, and selfgovernance. | - | Case study of Indigenous food sovereignty through a collaborative, multicommunity workshop in rural Interior Alaska. It is focused on defining and supporting food sovereignty through the lens of traditional harvest practices, cultural values, and Indigenous governance.  Actively advancing food sovereignty by participating in cultural activities such as revitalizing their language, maintaining sharing practices, and continuing to pass down knowledge of hunting and fishing practices to the next generation. |
| **“Community-led food resilience: A decolonizing autographic learning from an Inuit community”** [**https://doi.org/10.1016/j.gfs.2021.100564**](https://doi.org/10.1016/j.gfs.2021.100564) | | | | | | |
| Datta, R.  (2021)^(5)^ | Autoethnographic study | Inuit community in Northern Canada | ‘Food sovereignty is the right of Inuit to define their own hunting, gathering, fishing, land and water policies; the right to define what is sustainable, socially, economically and culturally appropriate for the distribution of food and to maintain ecological health; the right to obtain and maintain practices that ensure access to tools needed to obtain, process, store and consume traditional foods.’  ‘The term food sovereignty refers to Inuit ways of life, their sacred meanings, collaborative work, self-determination and self-governance, land rights, responsibilities, and collective decision-making.”  ‘Inuit food sovereignty is their sacred responsibility to “maintain healthy relationship with our land, plants, and animals that provide us with our food” | - Land, water, culture, and language rights  - Holistic perspective  - Ways of life  - Sacred  - Collaborative decision-making  - Self-determination and self-governance  - Natural law  - Intergenerational knowledge  - Elders and Knowledge Keepers’ teachings  - Food storage  -5Rs: “Relationships, Responsibility, Relevance, Respect, Relearn” (community-led food security) | The study challenges the use of “western scientific tools” in general and emphasizes relational learning, Elder/Knowledge Keeper guidance, and community-led knowledge as valid and scientific. | - |
| **“Exploring food sovereignty among Amazonian peoples: Brazil's national school feeding programme in Oriximiná, Pará state”** [**https://doi.org/10.1080/03066150.2024.2310149**](https://doi.org/10.1080/03066150.2024.2310149) | | | | | | |
| Affonso et al. (2024)^(6)^ | Community-based qualitative case study | Indigenous and forest-proximate communities in Oriximiná, Pará, Brazilian Amazonia | “Tropical forest food sovereignty: autonomously produced food that is shared among extended kin networks which also provide labour – with a centrality of women’s work and care for nature – in determinate territories *(Copeland 2019)* wherein local knowledge and agrobiodiversity are valued and reproduced intergeneration-ally”. | Five interrelated and mutually reinforcing dimensions linked by autonomy:  - Autonomous food production using extended family labour  - Sharing food among kin  -Valuing local origin and traditional production methods  - Avoidance of agrochemicals (for health and environmental reasons)  -Reproduction of local knowledge systems and agrobiodiversity intergenerationally |  | Indigenous families autonomously delivered traditional foods to schools; school meals included locally produced items like manioc and pumpkin; and quilombola youth organized protests demanding greater inclusion of family-farmed foods in public food programs (PNAE). |
| **“Indigenous food systems and food sovereignty: A collaborative conversation from the American Association of Geographers 2022 Annual Meeting” DOI: 10.5304/jafscd.2023.123.012** | | | | | | |
| Brant et al.  (2023)^(7)^  (Indigenous authors) | A synthesis and reflection of a collaborative panel session | Indigenous scholars and practitioners, primarily Mohawk (Kanyen’kehá:ka) from Tyendinaga and across North America/Turtle Island | “Indigenous food sovereignty is about moving away from a reliance on the market, and instead growing and harvesting food as part of relationships.”  “Indigenous food sovereignty is an approach to understanding how the regeneration of Indigenous food systems and practices contributes to decolonizing efforts, resisting state power, and achieving self-determination”. *(Daigle, 2019)* | - Relationship between all elements of creation - Working in relation to natural systems - Engaging with food from a deep level of respect - Involving ceremony and tradition - Learning and using Indigenous practices - Sharing foods across spaces and places as a way of sustaining and feeding oneself and the community | - | No formal case studies but multiple real-life practices are shared that can be considered *examples*:  . Reintroducing traditional methods of growing, preparing, eating, and preserving foods  . Harvest sharing with families, neighbors, and animals  . Community gardening and food sharing at FNTI  . Ceremony, land-based cycles, and seasonal rhythms  . Ancestral planting story (the Puebloan instructor example). |
| **“Examining Indigenous food sovereignty as a conceptual framework for health in two urban communities in Northern Ontario, Canada”** [**https://doi.org/10.1177/1757975919831639**](https://doi.org/10.1177/1757975919831639) | | | | | | |
| Ray et al.  (2019)^(8)^  (Indigenous authors) | Community-based participatory research | Northern Ontario, Canada | Indigenous Food Sovereignty (IFS) is a concept which embodies the importance of Indigenous peoples’ ability to control their food systems, including markets, production modes, cultures and environments and is essential for addressing this gap (4, p.2)  IFS moves beyond an individualized rights-based discourse to focus on the people of a territory and their responsibilities to and interdependence with place (42,6) and the resurgence of Indigenous foods and food systems (43,6) | Any examination of Indigenous people’s health and well-being must center the land, therefore IFS.  Interconnectedness, reciprocity, responsibility and respect  Key domains of IFS including policy and procedures, local food production and consumption, social justice and equity, local/cultural knowledges and practices, self-determination and governance, health of the land, and adaptability and resiliency. | - | A wild-food bank (e.g., distributing moose meat), annual medicine camps focused on traditional knowledge, community hunts for adults, youth, and families, and support for subsistence activities through licensing fee coverage.  A residential healing program grounded in land-based and ceremonial approaches, and water protection ceremonies such as the Annual Sudbury Anishnaabe-Kweg Water Walk. |
| **“Sogorea Te’ Land Trust empowers indigenous food sovereignty in the San Francisco Bay Area”** [**https://doi.org/10.5304/jafscd.2019.09B.003**](https://doi.org/10.5304/jafscd.2019.09B.003) | | | | | | |
| Wires K. N. et al.  (2019)^(9)^ | Case study | Chochenyo and Karkin Ohlone peoples (Oakland or the East San Francisco Bay Area of California) | ‘Indigenous food sovereignty is about much more than consumption choices, food access, and traditional knowledge; it is fundamentally about access to land for sacred ceremony and traditional practice.’ | - Access to Land  - Sacred space for ceremony  - Traditional Foodways and Ecological Knowledge  - Rematriation and Land Stewardship  - Healing and Wellness  - Community self-determination  - Land Governance  To restore native land stewardship to the original inhabitants of the land, to restore native foodways and traditional ecological knowledge, and to create sacred space for ceremony … to carry out their sacred obligations from their creator on the land. | - | The Sogorea Te’ Land Trust as a case study of Indigenous land rematriation in urban California. Daily life practices include planting traditional foods and medicines (e.g., acorns, basket-weaving plants), organizing ceremonies on protected land, building a traditional arbor and fire pit, and collaborating with Planting Justice to establish legal control over land use. |
| **“The Mauli Ola Study: A Unique Academic–Community Partnership With MA‘O Organic Farms to Understand and Address Health Inequities Among Native Hawaiians and Other Pacific Islanders in Hawai‘i” doi:10.1177/15248399231190356** | | | | | | |
| Maunakea et al.  (2023)^(10)^ | Community-based participatory research (CBPR) | Native Hawaiians and Pacific Islanders (NHPIs) in Wai‘anae, O‘ahu | - | Empowering a community food system that promotes traditional and modern foodways, reconnection with land (‘āina),  educational attainment for youth, health promotion, socioeconomic opportunities, and cultural restoration | - | The Youth Leadership Training (YLT) Program at MA'O Organic Farms provides an example of Indigenous food sovereignty. Youth participants engage in organic farming, reconnect with traditional food practices, and receive education on nutrition, health, and cultural values. Through their part-time work on the farm and educational experiences, they contribute to the local food system, providing fresh produce to families in their community while deepening their relationship with the land and reinforcing the concept of food sovereignty in daily life. |
| **“Food Sovereignty Indicators for Indigenous Community Capacity Building and Health”** [**https://doi.org/10.3389/fsufs.2021.704750**](https://doi.org/10.3389/fsufs.2021.704750) | | | | | | |
| Blue Bird Jernigan et al. (2021)^(11)^  (Indigenous authors) | Narrative Review | US and Canada | Indigenous food sovereignty, which extends the focus of food sovereignty in a number of ways, including primarily by emphasizing not only a community’s right but also their relational responsibilities to care for their food systems according to their traditional practices and beliefs *(Morrison, 2011; Coté, 2016).* | - Key indicators: access to resources, production, trade, food consumption, policy, community involvement, and culture  - Emphasizes the importance of culturally appropriate foods, community decision-making, sustainable food production, and control over food systems | - Indigenous Food Sovereignty Indicators (7 main indicators with 25 sub-indicators)  *- Food Sovereignty Assessment Tool (Bell-Shetter, 2004)*  *- Other food sovereignty assessment tools provide checklist style assessments, but do not provide guidance for direct action or a way to evaluate progress (Woodley et al., 2006; Binimelis et al., 2014; Weiler et al., 2014).* | - |
| **“Food sovereignty, food security and health equity: a meta-narrative mapping exercise” DOI:** [**10.1093/heapol/czu109**](https://doi.org/10.1093/heapol/czu109) | | | | | | |
| Weiler et al. (2015)^(12)^ | Meta-narrative review | Various regions, including Indigenous communities | Food sovereignty can be broadly understood as a call for people to have a greater capacity to ensure that farming, fishing, labour and land policies are appropriate to the diverse social and ecological contexts in which they occur *(Wittman 2011).*  Indigenous food sovereignty describes diverse indigenous communities’ ability to make decisions about their own consumption of healthful, culturally adapted indigenous foods, as well as their harvesting practices and relationships with the land *(Morrison 2011; Thompson et al. 2012; Rocha and Liberato 2013; Meyer 2014)* | - | Conceptual framework used to assess food system pathways to health equity, though no specific tools are listed. | Examples include the O-Pipon-Na-Piwin Cree Nation’s efforts to revive traditional foodways. The program supports community elders in passing on knowledge of traditional land-based livelihoods to youth, ensuring both cultural continuity and access to a healthy diet. It trains youth in hunting, fishing, community food-sharing, and other traditional skills. Simultaneously, the program addresses larger struggles for decolonization, self-reliance, participatory decision-making, and sustainable community economic development.  Six Peruvian Quechua communities collaboratively manage approximately 600 varieties of native potato to ensure the region’s food sovereignty by conserving its agrobiodiversity and traditional farming systems (CIP n.d.). |
| **“Blackfeet innovation pathways to food sovereignty: sustainability through indigenous-led research partnerships”** [**https://doi.org/10.3389/fsufs.2024.1303408**](https://doi.org/10.3389/fsufs.2024.1303408) | | | | | | |
| Paul, K. L. et al.  (2024)^(13)^ | Community case study | Amskapi Piikan Blackfeet Nation in northwestern Montana, United States | Piikani food sovereignty–the ‘whole’ represented by the graphic’s central circle and its overlapping fields of health and wellbeing, access to food and land, agriculture, and culture and traditional foods–connects priorities of the community to the land itself along with its layers of political and legal obstacles and opportunities (the yellow blocks at the bottom of the graphic), on the one hand, and to a selected list of strategic plans, research tools and tactics as well as a growing network of capacity building institutions, on the other. | - Culture and Traditional Foods  - Agriculture  - Health and Wellbeing  - Food & Land Access  - Sustainable economic development  - Cultural knowledge (Ihkitsiika)  - Legal rights and land  - Self-governance and participation | -  Piikani Well-being Index: focused on land ownership, land use, and elements of food sovereignty grounded in a community’s relationship to land and place | Blackfeet Innovation Pathways to Food Sovereignty project is a case study of IFS in action, embedded in land, culture, legal rights, and health.  A 100-day traditional diet study involving Piikani participants reclaiming ancestral food practices, and grazing practices rooted in Piikani stewardship values (e.g., Iinnii rotation).  An advisory council of Indigenous educators and food practitioners met monthly to guide the creation of a regional Indigenous-led food systems education and research initiative, resulting in a shared vision to support Indigenous food systems and proactively build capacity for Indigenous food sovereignty through collaborative approaches. |
| **“Traditional Food Upskilling as a Pathway to Urban Indigenous Food Sovereignty: Final Report”** [***https://uakn.org/wp-content/uploads/2015/05/21-UAKN-Research-Brief-Traditional-Food-Upskilling-as-a-Pathway-to-Urban-Indigenous-Food-Sovereignty_Final-.pdf***](https://uakn.org/wp-content/uploads/2015/05/21-UAKN-Research-Brief-Traditional-Food-Upskilling-as-a-Pathway-to-Urban-Indigenous-Food-Sovereignty_Final-.pdf) | | | | | | |
| Cidro et al.  (2016)^(14)^ | Qualitative, community-based research | Indigenous Peoples of the North End of Winnipeg | “IFS is important tool to combat food insecurity especially in urban centers as inner cities are known for having food deserts, and access to cultural food is limited to trade networks and a small number of market providers.” | IFS is guided by four main principles:  1. The recognition that food is sacred  2. Participation in food systems  3. Self-determination  4. Supportive legislation and policy | - | Workshops and feasts teaching traditional food skills; practicing culture in the city (e.g., fishing, growing foods); intergenerational knowledge transfer; reawakening of “food memory”; and food as a means of building relationships among participants. |
| **“Understanding Indigenous Food Sovereignty through an Indigenous Research Paradigm”** [**https://journalhosting.ucalgary.ca/index.php/jisd/article/view/58469**](https://journalhosting.ucalgary.ca/index.php/jisd/article/view/58469) | | | | | | |
| Martens et al.  (2016)^(15)^  (3 Indigenous authors and 1 non-Indigenous) | Qualitative, community-based participatory research (CBPR) | First Nations communities in Western Canada, across British Columbia, Alberta, Saskatchewan, and Manitoba. | “Indigenous food sovereignty has been described as a living reality for Indigenous people in Canada and refers to a re-connection to land-based food and political systems *(Morrison, 2011; Martens, 2015).*”  The interconnections among culture, heritage, spirituality, and politics, can make Indigenous food sovereignty difficult to define.” | Four principles of Indigenous food sovereignty that the Indigenous Food Systems Network has proposed:  1. Food is sacred and should be treated as such  2. Participation in land-based food activities  is important and requires an action-based approach  3. Self-determination of food systems is critical  4. Policy reform is a necessary component to addressing and achieving Indigenous food sovereignty goals.  *(Working Group on Indigenous Food Sovereignty, 2011).*  - Responsibility to land systems - Interdependent relationships with land, plants, animals, and people - Cultural, spiritual, and intergenerational knowledge-sharing | *-* | Examples include youth participating in gardening and carpentry for Elders in the Ladybug Garden (BC); the Back to the Land Camp in Peguis First Nation (MB); forest harvesting and wildcrafting in BC; and teachings from Elders at the Vancouver Island Traditional Foods Conference. These cases illustrate hands-on land-based activities, cultural and spiritual food practices, and intergenerational knowledge-sharing. |
| **“INDIGENOUS FOOD SOVEREIGNTY A Model for Social Learning”** | | | | | | |
| Morrison  (2011)^(16)^  (Indigenous author) | Theoretical model and principles for Indigenous food sovereignty | Indigenous communities in Canada, particularly British Columbia (B.C.) | “The concept of Indigenous food sovereignty describes, rather than defines, the present day strategies that enable and support the ability of Indigenous communities to sustain traditional hunting, fishing, gathering, farming and distribution practices (Morrison 2006, 2008), the way we have done for thousands of years prior to contact with the first European settlers.”  “Indigenous food sovereignty provides a restorative framework for health and community development and appreciates the ways in which we can work together cross-culturally to heal our relationships with one another and the land, plants and animals that provide us with our food.” | Four main principles of Indigenous Food Sovereignty:  -Sacred or Divine Sovereignty: Food is a gift from the Creator, and the right to food is sacred, beyond the jurisdiction of colonial laws.  -Participation: Continued active engagement in food-related activities at all levels (individual, family, community) is vital for maintaining food sovereignty.  -Self-Determination: Indigenous communities must have the freedom to respond to their own food needs through traditional harvesting and farming practices.  -Legislation and Policy: Indigenous food sovereignty seeks to reconcile traditional food values with colonial policies, aiming for policy reform in areas like forestry, fisheries, and health. | - | -R. v. Sparrow (1990): Recognized the right of the Musqueam Nation to fish for food, social, and ceremonial purposes, establishing precedence for Indigenous rights over traditional food practices.  -R. v. Gladstone (1996): The Heiltsuk First Nation's right to trade herring spawn for commercial purposes was recognized, further supporting Indigenous control over food systems.  -Delgamuukw v. British Columbia (1997): This landmark case affirmed that Aboriginal title includes the right to use the land for traditional purposes, such as hunting and fishing.  -Haida Nation v. British Columbia (2004): Required the Crown to consult Indigenous nations regarding the use of their traditional lands, which has implications for protecting food systems and resources.  Nuu-chah-nulth Fisheries Case (2009): The Supreme Court of British Columbia ruled that the Nuu-chah-nulth Nation has the right to commercially harvest and sell all species of fish within its traditional territorial waters.  Secwepemc community’s resilience during the Great Depression, where they used traditional food systems and knowledge to sustain themselves and provide food for both Indigenous and settler communities during a time of economic hardship. This example underscores the strength of traditional food systems and the role they play in community survival and resilience. |
| **“We Story the Land: Exploring Mi'kmaq food sovereignty, Indigenous law and treaty relations”** [**https://doi.org/10.1080/03066150.2023.2223482**](https://doi.org/10.1080/03066150.2023.2223482) | | | | | | |
| Stiegman & Pictou (2024)^(17)^  (One Indigenous and one non-Indigenous author) | Case study and documentary project exploring Mi’kmaq food sovereignty | Mi’kmaq community of L’sɨtkuk (Bear River First Nation), Kespukwitk, Atlantic Canada | “Indigenizing food sovereignty entails centering Indigenous political and legal orders, a move which fundamentally challenges Eurocentric, rights-based formulations of sovereignty (Coté 2016; Grey and Patel 2015); reveals the ﬁction of Lockean notions of private property and delegitimizes claims of settler-colonial state sovereignty (Daigle 2019; Kepkiewicz and Dale 2019). In this way, Indigenous food sovereignty unlocks what Grey & Patel refer to as ‘the radical potential of food sovereignty as a decolonizing activity’ (2015, 442), one that not only challenges the global corporate food regime but oﬀers a profoundly diﬀerent vision for how to organize social and ecological relations in nonexploiting ways.”  “Indigenous food sovereignty provides a framework for Indigenous resurgence to interface with other social movement sand to impact colonial policy (Morisson 2011).”  “Martens et al. (2016) stress the holistic philosophy of Indigenous food sovereignty, that recognizes food as encompassing mental, emotional, spiritual and intellectual aspects of being.” | The principles of Netukulimk form the foundation of Mi’kmaq food sovereignty, including:  - Reciprocal Relations: Respecting relationships with land, water, plants, and animals.  - Sustainable Practices: Harvesting in ways that maintain ecosystem health and balance.  - Treaty Rights and Stewardship: Exercising treaty rights to hunt and fish, while asserting Mi’kmaq jurisdiction and care over their territories.  Morrison (2011) four overarching IFS principles:  - Food is Sacred  - Participation in Food Systems  - Self-Determination  - Policy and Legislation | - | - Seven Paddles Project: A community-led initiative that reclaimed traditional canoe routes and engaged youth in reconnecting with ancestral lands, fostering cultural ties and practical skills related to food gathering.  - Community-based food practices include food fisheries, moose hunting, and annual harvest gatherings, all rooted in the principles of Netukulimk and supported by community Elders. |
| **“Beyond Food Security: Understanding Access to Cultural Food for Urban Indigenous People in Winnipeg as Indigenous Food Sovereignty”** | | | | | | |
| Cidro et al.  (2015)^(18)^  (One Indigenous author) | Qualitative study, Community-based participatory research | Urban Indigenous community of Winnipeg, Manitoba, Canada | “Indigenous food sovereignty (IFS) has been described as a “living reality” for thousands of years however; colonial impacts and landscape changes have threatened traditional and local food systems resulting, in part, in high levels of food insecurity and a need to reconnect people to their food systems (Morrison 2011).”  “Indigenous food sovereignty (IFS) goes beyond food security in that it looks to reconnect people and their food systems.” | - Cultural Food as Ceremony: Engaging in food practices as a form of ceremony that honors traditional knowledge and spiritual connections to food.  - Connection to Land through Reciprocity: Maintaining cultural food relationships by exchanging and sharing traditional foods, even in urban contexts.  - Re-learning IFS Practices: Addressing food insecurity by re-teaching traditional food knowledge and practices to urban Indigenous youth and community members.  - Relationship Formation: Emphasizing connections among people, between people and food, and between people and the land.  Morrison (2011): IFS is guided by four main principles: the recognition that food is sacred; participation in food systems; self-determination; and supportive legislation and policy | - | - Feasting and Gathering Practices: Participants described preparing and sharing cultural foods during family gatherings and community feasts.  - Ceremony and Spiritual Connection to Food: Food preparation described as a spiritual process, including praying over ingredients and participating in sweat lodges, powwows, and Sundances.  - Urban Gardening and Community-Supported Agriculture (CSA): Participants engaged in urban gardening programs or joined CSAs to grow or access traditional foods.  - Bartering: Participants described bartering (e.g., exchanging dried fruit for rabbit meat or yarn for teachings) as a culturally meaningful practice. |
| **“Improving Indigenous Food Sovereignty through sustainable food production: a narrative review”** [**https://doi.org/10.3389/fsufs.2024.1341146**](https://doi.org/10.3389/fsufs.2024.1341146) | | | | | | |
| Rowe et al.  (2024)^(19)^  (Include 5 Indigenous authors) | Narrative Review | Eastern tribes in the US | “In an Indigenous context, food sovereignty encompasses broader ideas rooted in Indigenous values, including relationships of reciprocity and responsibility to the land *(Miltenburg et al., 2022).*”  “The concept of Indigenous Food Sovereignty emerged in response to a food system that does not adequately serve Indigenous communities *(Hipp and Shirl, 2015; Maudrie et al., 2021).*”  “The Indigenous Food Sovereignty movement asserts the right of communities to ownership of their own food systems in ways that are culturally meaningful and empowering *(Maudrie et al., 2021).*” | - Strengthening community food production  - Environmental stewardship  - Relationality, responsibility, and reciprocity *(Miltenburg et al., 2022)*  - Natural resources security  - Education  - Self-determination  - Resilience  -Policy  - Community connection and involvement  - Cultural use | One framework introduced in the Indigenous Food Sovereignty literature highlights seven indicators that address an Indigenous community’s capacity for food sovereignty. These indicators include:  1. Access to resources  2. Production  3. Trade  4. Food consumption  5. Policy  6. Community involvement  7. Culture  *(Jernigan et al., 2021)* | IFS is practiced through growing and harvesting traditional foods, cooking and sharing meals, educating youth, practicing land stewardship, and reclaiming and preserving seeds and ecological knowledge.  - MAʻO Organic Farms (Hawai‘i): Youth are trained in traditional agricultural practices.  - God’s Country Waimanolo (Hawai‘i): Families build backyard aquaponics systems modeled after the traditional *ahupua‘a* system.  - Baltimore American Indian Center (BAIC): Youth summer camp at a farm focused on ecosystem-based agriculture  - Chickahominy Indian Tribe – Eastern Division: Runs a community garden and composting program, organizes food sovereignty education and plans a native food forest.  - Seneca Nation – Food Is Our Medicine Project: Early childhood and school programs teach traditional growing practices.  - Micmac Farms (Maine): Started as a garden to fight food insecurity, now an 18-acre farm with a trout hatchery and greenhouse.  - Maple Syrup Harvesting (Meskwaki Nation, Midwest): Revival of traditional maple syrup collection led by tribal youth.  - Seeds of Renewal (Vermont): Restores Abenaki horticultural practices through gardening classes, seed banks, and ceremonies.  - Santee Sioux Reservation, Iowa: encouraging more families to garden, from skill demonstrations to nutrition classes, cooking workshops, and gifts of starter plants.  - Wind River Reservation, Wyoming: Community gardening where families grow their food to bolster food security and resilience. |
| **“Growing Community Sustenance: The Social Economy as a Route to Indigenous Food Sovereignty” DOI: 10.29173/cjnser535** | | | | | | |
| Sumner et al.  (2023)^(20)^ | A descriptive mapping study based on publicly available online information | Indigenous communities in Canada | “Indigenous food sovereignty not only asserts the right of Indigenous Peoples to choose their own foodways, but also involves the ability to exercise that right.”  “Indigenous food sovereignty offers a restorative framework for nurturing relationships with each other and with the culturally important plants, animals, and waterways that provide people with food *(Wadden, 2010).*”  “Indigenous food sovereignty understands food as sacred and part of a web of relationships with the natural world that sustains culture and community.” *Martin and Amos (2017, p. 209)* maintain that Indigenous food sovereignty holds a special significance for Indigenous Peoples because, even though the terminology itself is relatively new, the concept “speaks to issues that Indigenous peoples and communities have been struggling with for many, many generations.” | Indigenous food sovereignty is based on a set of underlying principles, all of which reflect Indigenous worldviews: 1) food is sacred; 2) participation (at the individual, family, community, and regional levels); 3) self-determination; and 4) legislation and policy reform *(Martin & Amos, 2017).*  *-* Decolonization  *-* Relationality: relationships between people, land, animals, and food, grounded in community and reciprocity. | - | - Meechim Inc. – Garden Hill First Nation (Manitoba): A social enterprise producing local food to increase food security and reduce dependence on the Northern Store. Includes youth training, farming.  - Hopedale and Rigolet Inuit Community Governments (Newfoundland and Labrador): Established community gardens, joined the Good Food Box program, and expanded a community freezer to distribute hunted meat to elders and low-income households.  - Chippewas of Nawash Unceded First Nation (Ontario): Operates a community food bank, especially important during the COVID-19 pandemic.  - Fourteen Communities in Northern Manitoba – Country Food Programs: Support hunting, gathering, and fishing for community-wide benefit. Participants link these programs directly to food sovereignty.  - Opaskwayak Cree Nation (Manitoba): Developed community gardens and a fruit tree orchard. Aims to promote healthy living and food sovereignty.  - Peguis First Nation (Manitoba): Created agricultural initiatives post-relocation, including large-scale community gardening. Recognized for advancing sustainability, food sovereignty, and reconciliation.  - O-Pipon-Na-Piwin Cree Nation (South Indian Lake): Runs a country food service to support vulnerable members and re-skill hunters. Explicitly states food sovereignty as the program’s goal.  **- Northern Manitoba Food, Culture, and Community Collaborative (NMFCCC)**: A not-for-profit initiative supporting Indigenous-led food projects across northern Manitoba. Provides funding, training, and technical support for **community gardens**, **greenhouses**, and **country food programs**. |
| **“Our hands at work: Indigenous food sovereignty in Western Canada”** [**https://doi.org/10.5304/jafscd.2019.09B.007**](https://doi.org/10.5304/jafscd.2019.09B.007) | | | | | | |
| Robin, T.  (2019)^(21)^  (Indigenous author) | Qualitative community-based participatory research using Indigenous research methodology | First Nations and Métis communities in British Columbia, Alberta, Saskatchewan, and Manitoba | “Indigenous food sovereignty takes many forms and engages in spaces beyond gardens and waters and land.”  “Indigenous food sovereignty is a living, breathing way of life found in the past and present and envisioned for future generations.”  “Indigenous food sovereignty embodies a caring quality that extends to the land, water, and each other. IFS embraces an awareness of the intimate connection between people and all of creation.”  “Indigenous food sovereignty, as a concept and way of life, is challenging to describe and even more difficult to define.”  “Indigenous food sovereignty is a journey, and it is both ongoing and evolving.”  “Indigenous food sovereignty is a historical and lived experience that, in many cases, has been dismantled by colonialism.” | Four key elements of Indigenous food sovereignty that emerged from this research: history, connection to the land, relationships, and identity.  Additional principles include self-determination, the sacredness of food, and the importance of returning to the land to nourish both body and spirit—critical for well-being. *(Martens et al., 2019; LaDuke, 2005; Morrison, 2011; PFPP, 2011)* | - | Multiple case studies across 24 initiatives in Western Canada. Examples include: community gardens (Turtle Mountain Métis), land-based camps (Peguis First Nation), traditional food distribution (Nelson House), agroforestry (Splatsin), and cultural food education programs (T'Sou-ke Nation, Vancouver Island). Daily life practices include harvesting, gardening, food sharing, language transmission, and intergenerational learning. |
| **“Conceptualising the relationships between food sovereignty, food security and oral health among global Indigenous Communities: a scoping review”** [**https://doi.org/10.1017/s1368980024001198**](https://doi.org/10.1017/s1368980024001198) | | | | | | |
| Poirier et al.  (2024)(^22)^  (Indigenous and non-Indigenous authors) | Scoping review | First Nations, Métis, Inuit (Canada); Kaingang, Guarani (Brazil); Kichwa (Ecuador); Yalsih’on, Dzilam (Mexico); Aboriginal communities (Australia). | “Food sovereignty offers an alternative paradigm that recognises Indigenous Peoples’ right to self-determination and supports biodiversity conservation and diversified local food production systems *(Kepkiewicz L & Dale B, 2019)*. Indigenous food sovereignty, specifically, is understood as a movement that aims to reconnect Indigenous Peoples with their foodways.” | Four principles: (1) the recognition that food is sacred and the responsibility that Communities have in maintaining relationships with Lands, waters and beings; (2) daily Community participation in food systems; (3) Indigenous rights to self-determination and therefore the right to determine healthy and culturally relevant food systems and (4) the ability of Indigenous Communities to shape food-related policies. | - | Daily practices included maintaining traditional family farms (Kichwa, Ecuador), hunting and bush food gathering (Aboriginal communities, Australia), resisting processed food consumption, and preserving Indigenous foodways despite structural challenges (Kaingang and Guarani, Brazil; Yalsih’on, Mexico) |
| **“Framing an Indigenous Food Sovereignty Research Agenda”** [**https://doi.org/10.1177/15248399231190362**](https://doi.org/10.1177/15248399231190362) | | | | | | |
| Nguyen et al.  (2023)^(23)^  (Include Indigenous authors) | Conceptual study: research agenda | American Indian, Alaska Native, Native Hawaiian, and Pacific Islander communities across the United States | “Indigenous food sovereignty has grassroots origins, existing long before colonialism, with community members developing and leading programs to address their communities’ priorities. As initiatives grew, various conferences and summits have been hosted where leaders shared lessons learned with other interested communities.” | In 2021, Jernigan et al. identified seven key indicators supporting community capacity for Indigenous food sovereignty: access to resources, production, trade, food consumption, policy, community involvement, and culture *(Jernigan et al., 2021).* | - | - Tlingit region – traditional salmon harvesting: Traditional ecological management and food sovereignty through salmon stewardship (Langdon et al., 2006).  - Intergenerational learning and land stewardship: Daily life practices reinforcing food sovereignty through cultural education and place-based intergenerational knowledge exchange. |
| **“Indigenous food sovereignty is constrained by “time imperialism””** [**https://doi.org/10.1016/j.geoforum.2022.05.003**](https://doi.org/10.1016/j.geoforum.2022.05.003) | | | | | | |
| Ferguson et al.  (2022)^(24)^ | Qualitative, multi-sited, cross-case comparative study | Three coastal Indigenous populations, in Northwest Alaska, Palau, and Central Sulawesi | - | - Self-determination  - Intergenerational knowledge transfer  - Cultural and spiritual practices | - | - Inupiat communities (Kotzebue and Kivalina, Northwest Alaska): Harvesting bearded seal and caribou despite climate and work constraints; sharing subsistence foods; maintaining cultural practices tied to land and food.  - Sama Bajau and Suku Banggai communities (Banggai Archipelago, Central Sulawesi, Indonesia): Fishing and farming for local consumption; sending salted fish to children studying away; adjusting food practices during storm seasons (e.g., catching tibo-tibo fish). |
| **“Learning circles: an adaptive strategy to support food sovereignty among First Nations communities in Canada”** [**https://doi.org/10.1139/apnm-2021-0776**](https://doi.org/10.1139/apnm-2021-0776) | | | | | | |
| McEachern et al.  (2021)^(25)^ | Qualitative Community-based participatory research | Haida Gwaii (British Columbia); Hazelton/Upper Skeena (British Columbia); Ministikwan Lake (Saskatchewan); Black River (Manitoba) | “The core of the principles of food sovereignty is that Indigenous people should have control over what and how they eat.”  “Food sovereignty has an integral role, not only in food and nutrition security amongst Indigenous populations but as a path to Indigenous self-determination and reconciliation by non-Indigenous Canadians towards our First peoples.” | A review of conceptual frameworks linking Indigenous food sovereignty to health identified six practical “domains”, recognized by others as being important for effective development of food sovereignty in Indigenous communities:  - Policies and procedures  - Local food production and consumption & Local/cultural  knowledge and practice  - Self-determination and governance  - Social justice and equity  - Health of the land  - Adaptability and resiliency | - | Learning Circles: Local Healthy Food to School (LC:LHF2S): A community engagement model that aimed to enhance access to local, healthy, and traditional foods for youth.  Haida Gwaii: Development of local food pantries supplying traditional foods (e.g., salmon, berries, sea asparagus) to schools and hospitals.  Hazelton/Upper Skeena: Berry picking, fishing, and oolichan processing in schools guided by Elders.  Ministikwan Lake: School lunch program with traditional foods and student involvement in cooking. Elders-youth workshops, including the co-creation of a seasonal calendar. Permaculture.  Black River: Fishing, wild rice harvesting, and berry picking by school children. |
| **“Mobilizing Networks and Relationships Through Indigenous Food Sovereignty: The Indigenous Food Circle’s Response to the COVID-19 Pandemic in Northwestern Ontario”** [**https://doi.org/10.3389/fcomm.2021.672458**](https://doi.org/10.3389/fcomm.2021.672458) | | | | | | |
| Levkoe et al. (2021)^(26)^  (One Indigenous author and 2 non-Indigenous) | Community case study on Indigenous Food Circle's (IFC) response to COVID-19 | Indigenous communities in Northwestern Ontario, Canada | “Indigenous food sovereignty an ideal that builds on the right to healthy and culturally appropriate food and to ensure people have the power to determine and control their own food systems *(Patel, 2009).*”  “Beyond a rights-based discourse and settler conceptions of policy and governance, Indigenous food sovereignty focuses on the sacred connections among land and water-based food systems and the revitalization of traditional ecological knowledge, culture, and self-determination *(Morrison 2011; Coté, 2016; Whyte, 2017).*” | - Indigenous leadership and self-determination  -Sacred relationships with land and water  - Relationship Building and Networks  - Access to Traditional Foods  - Decolonizing Framework | - | The Northwestern Ontario Indigenous Food Sovereignty Collaborative (NOIFSC) launched the Sovereign Household Grant Program, which provided financial support for activities and equipment to increase food security capacity at the household level - advancing food sovereignty through direct, grassroots supports.  - The Indigenous Food Circle (IFC) supported multiple initiatives advancing Indigenous food sovereignty, including potato farming in Sandy Lake, installation of industrial fridges in Kasabonika, monthly fresh food boxes in Red Rock, and tools for hunting and fishing in several communities. |
| **“Enhancing cultural food security among the Syilx Okanagan adults with the reintroduction of Okanagan sockeye salmon”** [**https://doi.org/10.1139/apnm-2021-0321**](https://doi.org/10.1139/apnm-2021-0321) | | | | | | |
| Blanchet et al. (2022)^(27)^  (2 Indigenous authors and 3 non-Indigenous) | A cross-sectional, community-based participatory survey | Syilx Okanagan Nation, British Columbia, Canada | “For First Nations, reclaiming their food sovereignty, deﬁned as the right of Indigenous peoples to honour, value and protect their traditional food systems (Desmarais and Wittman 2014), is foundational to preserving their cultural food security, health, well-being, and way of life *(Power 2008; Kuhnlein and Burlingame2013; Council of Canadian Academies 2014; Amberson et al. 2016; Islam and Berkes 2016.*”  “Indigenous food sovereignty and cultural food security are interrelated, with the former considered as a necessary condition to achieve the latter *(Council of Canadian Academies 2014; Batal et al. 2021b).* Indigenous food sovereignty is based on the principle that decisions about Indigenous food systems should be made by Indigenous people *(Council of Canadian Academies 2014).*”  “Indigenous food sovereignty can be seen as a path towards decolonizing land, watersheds, and peoples as it emphasizes self-determination and the revitalization of Indigenous food systems, cultural practices and ecological knowledge systems *(Kamal et al. 2015, Grey and Patel 2015; Coté 2016).* ” | - Self-Determination  - Restoring cultural responsibilities and relationships to land, water, each other, and the natural world  - Decolonization of land, watersheds, and peoples  - Revitalization of Indigenous food systems and ecological knowledge  - Honouring, valuing, and protecting traditional food systems | - | - Syilx-led Reintroduction of Okanagan Sockeye Salmon into Skaha Lake: The Syilx Nation reintroduced sockeye salmon into Skaha Lake to support cultural food security and strengthen community connections. Through this program, the Syilx people engage in ceremonies, feasts, and traditional salmon harvests, reinforcing their cultural practices.  - Community Food Distribution: The Okanagan Nation Alliance (ONA) distributes salmon to Syilx households, early childhood programs, and schools, fostering access to culturally significant foods and supporting cultural continuity.  School-Based Salmon Education Program (Fish in Schools – FinS): “The intervention also includes an education component, Fish in Schools (FinS), that allows children… to raise salmon fry in their classroom and release them at the Annual ONA Sockeye Fry Release Ceremony.”  Fishing Camps: Teach community members traditional fishing skills and knowledge-sharing from Elders and knowledge keepers. *“The ONA organized a fishing camp to overcome barriers in knowledge, skills and equipment in the first years of the intervention.”* |
| **“Indigenous values and perspectives for strengthening food security and sovereignty: learning from a community-based case study of *Misko-ziibiing* (Bloodvein River First Nation), Manitoba, Canada”** [**https://doi.org/10.3389/fsufs.2024.1321231**](https://doi.org/10.3389/fsufs.2024.1321231) | | | | | | |
| Young et al. (2024)^(28)^  (Indigenous authors) | Community-based qualitative case study | Misko-ziibiing (Bloodvein River First Nation), Manitoba, Canada | “Indigenous food sovereignty (self-determined food systems governance).”  “Indigenous food sovereignty can and will look different in every community. Expanding beyond Miskoziibiing, the concepts discussed in this article can provide guidance and examples of how embracing local and Indigenous food systems values can strengthen Indigenous governance and contribute to the ongoing health, wellness, and sustainability of communities and the lands and territories that surround them.” | - Connection to land: Guided by the values of relationality, responsibility, and reciprocity  - Intergenerational transmission: Knowledge of hunting, trapping, gathering, and food preparation passed through family and community  - Community support and sharing: Mutual aid through food sharing and collective support, involving local governance  - Working together: Emphasis on family and community collaboration for harvesting and teaching  - Language revitalization: Recognized as critical to food practices and cultural continuity  From Morrison (2011) and the Indigenous Food Systems Network: (1) food is sacred; (2) participation in land-based activities is essential; (3) self-determination; (4) and policy reform. | - | - Food Sharing and Reciprocity: Elders recall the importance of sharing harvested food with community members as a survival strategy and a cultural practice. One Elder explains that sharing was essential for survival, where “everything, from food to instruments, was shared among families”.  - Land-Based Teachings: The community’s Prevention Program helps pass down traditional food knowledge, involving youth in hunting, fishing, and gathering activities guided by Elders, promoting self-sufficiency and resilience among younger generations |
| **“Relationality, Responsibility and Reciprocity: Cultivating Indigenous Food Sovereignty within Urban Environments”** [**https://doi.org/10.3390/nu14091737**](https://doi.org/10.3390/nu14091737) | | | | | | |
| Miltenburg et al.  (2022)^(29)^ | Community-based participatory research with urban Indigenous community members | Indigenous community members in the Grand River Territory, southern Ontario, Canada | “IFS recognizes the sacred, participatory relationships between Indigenous Peoples and their foods, concretizing practices and knowledges that are shared to support Indigenous food systems *(Morrison 2011)*.”  “IFS is an action-oriented approach that supports these place-based food systems, through activities that consider the sociocultural meanings, acquisition, and processing techniques of Indigenous foods such as hunting, fishing, trapping, and foraging *Morrison 2020, Daigle 2017, Willows 2005).*” | The study identifies three relational principles as key elements of IFS:  - Relationality: Emphasizes interconnectedness among Land, food, community and Creator.  - Responsibility: Refers to community obligations to care for the Land and its resources.  - Reciprocity: Involves mutual exchange and sharing within the community, ensuring that all resources, including food, are distributed respectfully  - Self-determination is central to IFS, as community members actively decide what to grow, how to harvest, and how to share food, reclaiming control over their food systems. | - | - Seed Saving and Urban Gardens: Initiatives included seed saving, growing Indigenous foods, and distributing produce within the community, such as in the Wisahkotewinowak collective’s gardens, which offer Land-based learning and food-sharing opportunities.  - Food Sharing Programs: Indigenous food baskets and communal food-sharing initiatives were launched during the COVID-19 pandemic to support Indigenous families in urban areas, fostering connections and providing culturally significant foods |
| **“We Need to Live off the Land”: An Exploration and Conceptualization of Community-Based Indigenous Food Sovereignty Experiences and Practices”** [**https://doi.org/10.3390/ijerph20054627**](https://doi.org/10.3390/ijerph20054627) | | | | | | |
| Poirier and Neufeld  (2023)^(30)^ | Community-based participatory research (CBPR) using qualitative methods; Indigenous methodologies (Etuaptmum: Two-Eyed Seeing) | A First Nation community on Vancouver Island (Coast Salish Territory), British Columbia, Canada | “Indigenous Food Sovereignty (IFS) movements work to counteract the dismantling of Indigenous food systems as well as the negative health impacts of environmental dispossession experienced by Indigenous communities.”  “IFS strives to increase community control of Indigenous food systems as a platform towards self-determination, cultural reclamation *(Kamal 2015)* and restoring relationships *(Neufeld 2017).* IFS recognizes the ongoing impacts of colonization in communities and offers a human rights, land reform and self-determination approach because hunger is not the only impact a shift in food systems has for Indigenous communities *(Matties 2016, Grey 2015).*”  “IFS works to describe, rather than define, processes that support IK including hunting, gathering, growing, preparing and preserving traditional foods *(Wiebe 2011, Grey 2015).*” | The study identifies three key elements that underpin IFS:  1. Environmental Concerns: Participants voiced concerns about pollution, climate change, and loss of natural resources that threaten traditional food sources.  2. Sustainable Practices: IFS movements focus on sustaining traditional methods and knowledge to ensure the continuation of food sovereignty across generations.  3. Relationship with Land and Waters: Strengthening connections to traditional lands and resources, which participants identified as essential to cultural and spiritual health  Four principals *(Morrison)*:  1. The recognition that food is sacred  2. Participation in food systems  3. Self-determination  4. Policy | - | - Community Garden: The community garden is used to supply food for weekly luncheons, culture night dinners, and the Elder meals-on-wheels program. It also grows medicinal herbs, which are dried and distributed within the community.  - Traditional Workshops: Participants organize workshops on food preservation and smoking methods to reinforce Indigenous Knowledge and share skills within the community.  - Harvesting and Sharing: Community members gather traditional foods, replicating ancestral methods to teach youth. Fish harvesting and distribution are organized annually among households, ensuring community members retain access to culturally significant food. |
| **“A Recipe for Change: Reclamation of Indigenous Food Sovereignty in *O-Pipon-Na-Piwin Cree* Nation for Decolonization, Resource Sharing, and Cultural Restoration”** [**http://dx.doi.org/10.1080/14747731.2015.1039761**](http://dx.doi.org/10.1080/14747731.2015.1039761) | | | | | | |
| Kamal et al. (2015)^(31)^ | Case study of community-based food sovereignty program | O-Pipon-Na-Piwin Cree Nation (OPCN), Northern Manitoba, Canada | “Indigenous food sovereignty addresses ‘Indigenous’ aspirations for collective well-being and prefers to rectify inequality and acknowledge peoples’ rights to land and cultural integrity *(Morrison, 2011).*”  “Indigenous food sovereignty advocates for decolonizing activities—reclaiming land for hunting, fishing, trapping, berry picking, community gardens, wild food programs, and other cultural activities, along with their cultural meanings.”  Indigenous food sovereignty, which emphasizes the importance of cultural practices, is a pathway in this case towards decolonizing land and peoples. | The study identifies key cultural values central to OPCN's food sovereignty:  - Resource Sharing (*wechihituwin*): concept of resource. The term emphasizes the fact that food in the cultural tradition of OPCN is not a commodity; it is a set of relationships.  - Decolonization (*pasekonekewin*): taking the person by the hand and helping her or him stand. The term is used to elaborate the integration of youth in food harvesting activities.  - Youth Empowerment: Emphasizes intergenerational knowledge transmission through traditional practices, such as hunting, fishing, and berry picking, which are integrated into educational programs | - | - Ithinto Mechisowin Program (IMP): This “Food from the Land” program provides wild foods to community members in need, aiming to restore traditional food practices, cultural knowledge, and community resilience.  - Youth Empowerment through Land-Based Education: The program collaborates with the local school to teach youth Cree values related to food, such as respect, sharing, and responsibility, reinforcing cultural connections and intergenerational knowledge transmission.  - Storytelling in Cree during Harvesting Activities: Stories are shared during land-based practices, with teachings such as: “Each food harvested from the land has a story that teaches something to us”. |
| **“Where Creator Has My Feet, There I Will Be Responsible”: Place-Making in Urban Environments through Indigenous Food Sovereignty Initiatives”** [**https://doi.org/10.3390/ijerph20115970**](https://doi.org/10.3390/ijerph20115970) | | | | | | |
| Miltenburg et al.  (2023)^(32)^  (Include Indigenous authors) | Qualitative exploratory study using community-based participatory research | Urban Indigenous communities in the Grand River Territory (Kitchener, Waterloo, Guelph, and Cambridge), southern Ontario, Canada | “Fostering relationships with Land is a starting point towards IFS and Indigenous self-determination.”  “IFS requires a return to the cultural values and ways of knowing about food, which can be achieved through processes of environmental repossession (Richmond et al., 2020).”  “Responsibilities are at the core of food sovereignty to ensure food security, which is understood and carried on through ceremonies to respect the spiritual aspects of seed keeping, planting, and caring for the Land (Delormier et al., 2017; Delormier et al., 2019).” | Key principles highlighted in the study include:  - Reciprocity – Recognizing that Land and food are gifts that must be respected and treated with gratitude.  - Relationality – Emphasizing the interconnectedness between Land, people, plants, animals, and ancestors.  - Responsibility – Highlighting the obligation to care for Land, community, and food systems, rooted in Indigenous worldviews.  - Self-determination and access to Land – Asserting the right to govern food systems and engage in Land-based practices, even within urban environments. | - | - Wisahkotewinowak Collective: Operates multiple community gardens that serve as spaces for cultivating Indigenous foods and engaging in Land-based practices.  - Waterloo Region Indigenous Food Sovereignty Collective: Applied a grassroots, neighborhood-based approach to food sharing and meal making, involving backyard gardeners and a larger seed garden to support community food needs.  - White Owl Native Ancestry Association – Food Share Program: Partnered with Indigenous student centers and the Wisahkotewinowak Collective to distribute food through preserving workshops and food baskets.  - North End Harvest Market: provided culturally significant foods to the local Indigenous community by sourcing foods from various Indigenous suppliers. |
| **“Indigenous food sovereignty in a captured state: the Garifuna in Honduras”** [**https://doi.org/10.1080/01436597.2020.1768840**](https://doi.org/10.1080/01436597.2020.1768840) | | | | | | |
| MacNeill (2020)^(33)^ | Qualitative, ethnographic study | Garifuna communities along the North Coast of Honduras | “Indigenous food sovereignty movements disrupt mainstream, Western development ‘myths’ as ‘groups repeatedly demonstrate … their initiative, creativity, and resistance while maintaining subaltern languages and identities’ (Radcliffe 2012, 92). They also emphasise self-determination over the ways in which needs for healthy, culturally relevant foods are met (Desmarais and Wittman2014). This involves calls for legislative and policy reform that creatively attempt to ‘reconcile Indigenous food and cultural values with colonial laws, policies and mainstream economic activities’ (Morrison 2011, 101).”  “Garifuna food sovereignty may engage and contest a different (although overlapping) set of power entanglements than does that of its Mestizo Honduran counterparts, making the concept of ‘sovereignty’ more salient than that of ‘security’.” | The study highlights core principles driving Garifuna food sovereignty:  - Self-determination and autonomy – *“We need to produce to bring autonomy and the sovereignty of our peoples.”*  - Connection and access to land – Emphasizing the importance of land for cultural survival, food production, and community well-being.  - Resistance to colonialism – Framing food sovereignty as a decolonial response to ongoing dispossession and neoliberal development pressures. | - | Land Reclamation as Food Sovereignty: Garifuna resistance to foreign-led tourism projects, such as the Banana Coast cruise port, reflects efforts to reclaim traditional lands crucial for food production and cultural continuity.  Subsistence Practices and Land Defense: In areas like Chachahuate, Garifuna continue practicing subsistence fishing and farming. In other areas, land occupations and resistance efforts demonstrate how food sovereignty is lived as a daily political and survival strategy. |
| **“Enhancing Indigenous food sovereignty: A five-year collaborative tribal-university research and extension project in California and Oregon”** [**https://doi.org/10.5304/jafscd.2019.09B.013**](https://doi.org/10.5304/jafscd.2019.09B.013) | | | | | | |
| Sowerwine et al.  (2019)^(34)^ | Mixed-methods study using a Community-Based Participatory Research (CBPR) approach within a tribal–university collaboration | Karuk, Yurok, and Klamath Tribes in the Klamath River Basin, California, and Oregon, USA | “Indigenous food sovereignty, “refers to a re-connection to land-based food and political systems” *(Martens, Cidro, Hart, & McLachan, 2016)* and seeks to uphold “sacred responsibilities to nurture healthy, interdependent relationships with the land, plants, and animals that provide us with our food” *(Morrison, 2011).*”  “The concept of Indigenous food sovereignty is not just focused on *rights* to land and food and the ability to control a production system, but also *responsibilities* to them, which encompasses culturally, ecologically, and spiritually appropriate *relationships* with elements of those systems *(Hoover, 2017).*” | - Self-determination – Leadership and control over food systems and research.  - Intergenerational knowledge transmission – Passing down knowledge and practices.  - Decolonization – Reclaiming food systems from colonial structures.  - Land stewardship – Caring for land through traditional ecological practices.  - Cultural values and identity – Strengthening identity through food, language, and ceremony.  - Youth empowerment and community health – Supporting well-being through cultural practices. | - | - Ishkêesh’tunviiv (River Kids) Program – An after-school program engaging Karuk youth in harvesting, food prep, cultural arts, and storytelling focused on Native foods.  Seasonal food camps and workshops –like acorn preparation, salmon canning, deer butchering, pit oven cooking, camas digging, and tule mat weaving.  - Píkyav Field Institute – a tribal-led research, education, and workforce development institute; and the integration of cultural values into extension through workshops and seasonal food camps.  - Native curriculum development – The *Nanu’ávaha* (“Our Food”) K-12 curriculum, adopted by local schools, incorporates tribal food systems and cultural knowledge into classroom learning.  - Master Gardener training for Klamath Tribal members – Empowered community members to grow and teach others about gardening and food security. |
| **“Food Sovereignty in Indigenous Communities: Extension Programs for Health, Culture, and Resilience”** [**https://doi.org/10.34068/joe.61.03.04**](https://doi.org/10.34068/joe.61.03.04) | | | | | | |
| Hartmann, K. (2023)^(35)^ | Qualitative interview study | Western region of Extension (Alaska, Arizona, California, Colorado, Hawaii,  Idaho, Montana, Nevada, New Mexico, Oregon, Utah,  Washington, and Wyoming), American Samoa, Gaum,  Micronesia, and the Northern Mariana Islands. | “For Indigenous communities, food sovereignty is part of the larger struggle for political, cultural, and ecological sovereignty *(Ruelle, 2017);* it has been applied to Indigenous rights movements *(Claeys, 2012).*”  “Indigenous rights movements use the idea of food sovereignty in a way that, “involves the relationship between a physical territoriality and a kincentric universe.” By “kincentic,” they recognize that Indigenous peoples extend their social relations to the extant cosmos, a very different view than the commodification of capitalism. Therefore, “food can be seen as the most direct manifestation of the relationships between Indigenous Peoples and homelands, and it consequently occupies a central place in traditional thought”.” | - Political, cultural, and ecological sovereignty  - kincentric relationships to land and cosmos  - Cultural and health significance of traditional foods  - Autonomy and self-determination; | - | IFS through gardening, education, restoration of traditional species, cultural knowledge transfer, and urban food initiatives.  Food Sovereignty Gardens for Health and Autonomy: “They were interested in food sovereignty, so I started working with them on their food sovereignty gardens...”  Reviving Traditional Foods and Food Mapping`: “One of our FRTEP Agents... had a project where they map the old diets of the [Tribe].”  Salmon Restoration and Traditional Food Education in Schools: “The [Tribe] has been working hard... salmon release programs into our ancestral tributaries.” |
| **“Food Sovereignty and Health: A Conceptual Framework to Advance Research and Practice”** [**https://doi.org/10.1177/15248399231190367**](https://doi.org/10.1177/15248399231190367) | | | | | | |
| Blue Bird Jernigan et al.  (2023)^(36)^  (Indigenous author) | Conceptual framework article | American Indian, Alaska Native, Native Hawaiian, and Pacific Islander communities across the United States | “Food sovereignty… Indigenous communities expand on this concept to incorporate the relational and multidimensional nature of food and health.*”*  “Indigenous food sovereignty cultivates awareness, capacity, and skills that come directly from the community, are owned by the community, and can be used by the community to advocate for policies, funding, and support that align with the community’s goals.*”* | - Relationality and restoration of responsibilities to land, food, and people  - Intergenerational knowledge transmission  - Community ownership and self-determination  - Land-based practices (hunting, gathering, growing)  - Holistic, multidimensional health (physical, emotional, spiritual)  - Resistance to colonial structures and inequities | - | Hunting, gathering, gardening, land-based learning with Elders, youth programs to restore traditional foodways, and tribal farms. |
| **“Food insecurities and dependencies: Indigenous food responses to COVID-19”** [**https://doi.org/10.1177/11771801221137639**](https://doi.org/10.1177/11771801221137639) | | | | | | |
| Alabi et al. (2022)^(37)^  (1 Indigenous author) | Commentary | Indigenous communities in Canada | Indigenous food sovereignty offers a new pathway to address food security, by Indigenous peoples, for Indigenous peoples, and with Indigenous peoples *(Martens et al., 2016)*. However, there is a danger to limiting the focus of Indigenous food sovereignty as merely addressing food insecurity. Food, for many Indigenous peoples, is more than consumption.*”*  *Morrison (2011)* has argued that there is no singular definition of Indigenous food sovereignty, that Indigenous communities are unique and thus have their own approaches and practices towards food and the land. Similarly, *Daigle (2019)* has challenged us to consider food sovereignties rather than a singular idea of food sovereignty.*”*  Indigenous food sovereignty describes an epistemology, movement, and way of life in Canada whereby Indigenous communities and peoples are revitalizing Indigenous food systems.*”*  Indigenous food sovereignty provides a pathway towards long-term strategies for communities to build sustainable and living food stores and their associated cultural responses, such as ceremony, language, and traditional governance.*”* | Indigenous food sovereignty has been said to be  - local,  - relational,  - reciprocal,  - spiritual,  - responsibility-driven, and  - holistic *(Coté, 2016; Daigle, 2017; Morrison, 2020; Robin, 2019).* It contains spirit and is a way to provide nourishment for  mind, body, and spirit.  - Self-determination | - | - Nearly a quarter of the 360 houses in Skidegate are already gardening (Duchesne, 2020). This has helped to mitigate the impact of job losses caused by the pandemic.  - Three community gardens are thriving, with 75 fruit trees planted across the neighbourhood. Greenhouses are located in both elementary and high schools, and students bring plants home to their families. Everything from squash, tomatoes, and artichokes to medicinal plants and even potatoes are being grown by gardeners  - Ninety families receive a weekly food security box with wild and traditional items like deer sausage and halibut, as well as pantry essentials (Duchesne, 2020).  -Chipewyan Prairie First Nation and Fort McKay Métis community in Alberta are navigating the COVID-19 pandemic by leveraging their relationships with local companies to make food available for their people. |
| **“Relative to the landscape: Producer cooperatives in native food sovereignty initiatives”** [**https://doi.org/10.1016/j.jcom.2021.100147**](https://doi.org/10.1016/j.jcom.2021.100147) | | | | | | |
| Dower & Gaddis  (2021)^(38)^ | Qualitative, multi-method case study | Indigenous communities in the United States | “Indigenous food sovereignty incorporates the place-based, and cyclical understandings of Native communities in efforts to rebuild culturally appropriate food systems and mitigate the efforts of colonial diets on physical and mental health. Furthermore, because most tribal food expenditures support non-Native food producers, Indigenous food sovereignty activists see reclaiming foodways as an opportunity to re-localized food economies through tribally led, place-based food initiatives *(LaDuke, 2008).”*  “Indigenous food sovereignty, recognizes the unique traditional knowledge, innovations, and land-use practices of Native foodways *(Grey & Patel, 2015; Rudolph & McLachlan, 2013; Wiebe & Wipf, 2011)*.” | Food sovereignty thus aspires to autonomy, social justice, and balance with the rest of nature, while offering a pathway to recenter food systems with intergenerational consciousness and action.  The Working Group on Indigenous Food Sovereignty:  1. Indigenous food systems recognize food is sacred and from the Creator, and therefore  comes with responsibilities to uphold reciprocal relationships to the ecological community.  2. Foodways are participatory.  3. Indigenous food practices encourage self-determination and the right to be free from the corporate control and oppressive policies and practices around land, water and social structures.  4. Indigenous food sovereignty calls for supportive legislation and policy. | - | Four case studies of community-led cooperatives:  Ohe·láku White Corn Cooperative (Oneida Nation, Wisconsin): Community members collectively plant, harvest, and process Haudenosaunee White Corn.  San Xavier Cooperative Farm (Tohono O’odham Nation, Arizona): Grows traditional crops such as cholla buds, heirloom beans, and drought-resistant corn.  Seminole Beef Cooperative (Florida): Tribally owned cattle cooperative. Beef is sold to institutions, including school lunch programs.  Intertribal Maple Syrup Cooperative (Great Lakes Region): Supports maple syrup production in sugar camps rooted in Anishinaabe teachings. |
| ***“Food Security and Food Sovereignty: The Difference Between Surviving and Thriving”*** [***https://doi.org/10.1177/15248399231190366***](https://doi.org/10.1177/15248399231190366) | | | | | | |
| Maudrie et al. (2023)^(39)^  (Indigenous authors) | Literature review with community perspectives on Indigenous food security and food sovereignty | American Indian and Alaska Native (AI/AN) communities across the United States | Indigenous food sovereignty is “a holistic approach to food that incorporates values of relationality, reciprocity, and relationships. Fundamental differences exist between food security and food sovereignty, yet dominant society often reduces IFS as a solution to food security, rather than an entirely different food system that is predicated on values that contrast with that of dominant society.”  “Indigenous conceptions of food sovereignty expand on the values offered through the broader food sovereignty movement (e.g., agency, rights-based approach to food) by centering the value of food to Indigenous peoples in holistic well-being (physical, social, spiritual, emotional, and intellectual), in being accountable relatives to our food systems, and emphasizing responsibility to engage with one another and our food systems with reciprocity and respect *(Martens et  al., 2016)*. ” | The key elements central to Indigenous food sovereignty include:  - Relationality: Engaging in practices that honor the interconnections between people, land, and all living beings.  - Reciprocity: Emphasizing mutual exchange and care within food systems.  - Relationships: Maintaining respectful and accountable relationships with more-than-human relatives (land, plants, animals) | - | - Community Initiatives: “Nikolaus et al. (2022) highlights many community-led interventions that center around the revitalization and cultivation of culturally relevant foods. This includes a tribally owned and operated farm, and the transmission of intergenerational knowledge related to traditional foods, their cultivation, processing, and preparation.” |
| **“An Indigenous food sovereignty initiative is positively associated with well-being and cultural connectedness in a survey of Syilx Okanagan adults in British Columbia, Canada”** [**https://doi.org/10.1186/s12889-021-11229-2**](https://doi.org/10.1186/s12889-021-11229-2) | | | | | | |
| Blanchet et al. (2021)^(40)^  (Two Indigenous authors and 4 non-Indigenous) | Cross-sectional Survey of Syilx Okanagan adults | Syilx Okanagan Nation, British Columbia, Canada | Indigenous food sovereignty describes the right of Indigenous Peoples the right of Indigenous peoples to practice ancient cultural activities such as hunting, fishing, trapping, plant harvesting, and agriculture in certain areas to sustain themselves.” *(Morrison, 2011)* It is seen as essential to “decolonizing land, water, and people” by privileging “Indigenous worldviews, livelihoods, and governance” *(Delormier et al, 2017)* | Key elements highlighted include:  - Self-determination and Rights to Traditional Practices: Emphasis on the right to harvest, process, and consume culturally significant foods such as salmon.  - Cultural Resilience and Connection to Land: The initiative is seen as a way to restore traditional food sources and cultural continuity.  - Community Health and Well-being: Participation in the salmon reintroduction initiative is associated with improved cultural connectedness and well-being among community members | - | - Sockeye Salmon Reintroduction: A Syilx-led initiative to restore Okanagan Sockeye salmon populations. The project involved habitat restoration, traditional ceremonies, and fish fry releases to re-establish salmon as a key cultural and food source.  - Cultural Practices and Feasts: Community members participate in salmon feasts, ceremonies, and traditional food practices, reinforcing cultural bonds and promoting well-being |
| **“Oil pipelines and food sovereignty: threat to health equity for Indigenous communities”** [**https://doi.org/10.1057/s41271-019-00186-1**](https://doi.org/10.1057/s41271-019-00186-1) | | | | | | |
| Jonasson et al. (2019)^(41)^  (One Indigenous author) | Case study focused on environmental health impact from a pipeline expansion project | Tsleil-Waututh Nation (TWN), British Columbia, Canada | Indigenous ‘food sovereignty’—  local peoples’ self-determined, inherent, never-ceded right to control their own local  food systems, ensuring that gathering practices, ecological resources, and policies  for labour and land are appropriate and relevant to their local contexts *(Wittman, 2011; Weiler et al, 2015).* | Core elements include:  - Self-determination and Autonomy: Control over traditional food resources and practices.  - Environmental Stewardship: Protection against contamination from industrial projects to safeguard traditional food sources like shellfish.  - Health and Cultural Continuity: Preserving traditional food access for physical, mental, and cultural well-being. | - | - Shellfish Harvesting and Restoration: TWN’s Burrard Inlet Action Plan focuses on reducing contamination to restore traditional shellfish harvesting, integral to TWN’s cultural practices.  - Environmental Monitoring: TWN’s monitoring of biotoxins and pollutants in shellfish and water demonstrates their efforts to protect food sovereignty and health amidst environmental threats from pipeline expansion. |
| **“Food Sovereignty in Whaingaroa: Perspectives of Food Providers in a Small, Coastal New Zealand Township”** [**http://dx.doi.org/10.1080/00664677.2016.1190920**](http://dx.doi.org/10.1080/00664677.2016.1190920) | | | | | | |
| Ritchie, I.  (2016)^(42)^  (Indigenous author) | Qualitative ethnographic research | Whaingaroa, central North Island, New Zealand | -  IFS is illustrated through the Hua Parakore initiative: “Hua Parakore is a development opportunity in the form of an indigenous food sovereignty initiative that seeks to address and respond to this triple crisis from a kaupapa Māori [Māori philosophical] framework and assert rangatiratanga [self-determination] with regard to food production.”  “Food sovereignty also values and supports the contributions of indigenous people.” | - Rangatiratanga (Self-determination)  - Whakapapa (Ancestral connection to land and food)  - Wairua and Mauri (Spirituality and life force)  - Manaakitanga (Care and respect)  - Kaitiakitanga (Guardianship) | - | - Hua Parakore: An Indigenous certification system and food sovereignty initiative grounded in kaupapa Māori principles and resisting GM, biopiracy, and commodification.  - Te Waka Kai Ora: National Māori organics organization supporting local producers.  - Community gardening, land sharing, WWOOFing (‘Willing Workers on Organic Farms’, who exchange their labour for accommodation and food), and sustainable farming practices. |
| **“Food sovereignty and autonomy for indigenous health as resistance to food globalization: Scoping review”** [**https://doi.org/10.1016/j.gfs.2023.100682**](https://doi.org/10.1016/j.gfs.2023.100682) | | | | | | |
| Farfan et al. (2023)^(43)^ | Scoping review | Various global Indigenous contexts | “Indigenous food sovereignty advocates for decolonizing activities to restore and develop cultural practices, values, and thoughts.”  “…The practice of indigenous food sovereignty using local food harvesting programs can provide an opportunity for decolonization in Canada (Kamal et al., 2015; Blanchet et al., 2021).” | - Self-determination and autonomy  - Cultural and Environmental Sustainability  - Decolonization and resistance to Globalization  *- Buen Vivir: Inspired by Indigenous worldviews, promoting coexistence with nature, sustainability, and equity as an alternative to conventional development models. The paper references the Quechua and Maori philosophies of Buen Vivir as expressions of sovereignty in food practices.* | - | - Use of Indigenous seeds; planting, harvesting, and exchange of local foods; wild food programs; and food practices grounded in Indigenous cosmovision and life plans. |
| **“Beyond culinary colonialism: indigenous food sovereignty, liberal multiculturalism, and the control of gastronomic capital”** [**https://doi.org/10.1007/s10460-018-9868-2**](https://doi.org/10.1007/s10460-018-9868-2) | | | | | | |
| Grey & Newman (2018)^(44)^ | Theoretical and conceptual paper | Indigenous communities in Canada and Peru | “Food sovereignty for Indigenous Peoples asserts the imperative of and both the right and responsibility to cultivate/hunt/gather traditional foods on traditional lands, to nurture Indigenous cultural practices around food preparation, consumption, and storage, and to decolonize the local and national food culture to augment both Indigenous health outcomes and cultural resurgence *(Grey and Patel 2015).*”  “Supporting Indigenous  food sovereignty, by definition, entails affirming the inherent  and treaty rights under which Indigenous Peoples may  cultivate, hunt, gather, steward, *and prepare, serve, and eat*  traditional foods *on traditional territories*.”  “Food systems justice, and the very definition of food sovereignty, thus requires recognition of Indigenous Peoples’ right to hold gastronomic capital back from the market.” | - Right and responsibility to cultivate/hunt/gather traditional foods on traditional lands  - Active sharing and mindful withholding of food and cuisine  - Self-determination  - Governance and Stewardship  - Right to withhold gastronomic capital from the market  - Decolonization | - | - Resistance to Gastronomic Multiculturalism: The paper discusses how Indigenous communities in Canada and Peru resist culinary appropriation in national and international markets, emphasizing food sovereignty as a means to combat culinary colonialism and protect cultural values.  - Canada: Salmon stewardship, potlatch, FSC fishing rights, and co-management practices in Pacific Northwest First Nations.  - Peru: Quechua-led efforts to reclaim control over native crops (e.g., potatoes and kinwa), legal advocacy, and protection of biocultural heritage. |
| **“Restorying Northern Arapaho food Sovereignty”** [**https://doi.org/10.5304/jafscd.2019.09B.012**](https://doi.org/10.5304/jafscd.2019.09B.012) | | | | | | |
| Arthur & Porter  (2019)^(45)^  (Indigenous author) | Narrative-based community case study | Northern Arapaho, Wind River Reservation, Wyoming, USA | -  “By reclaiming our food sovereignty, Indigenous nations are also restoring our identities, cultures, his/stories, and traditions.” | - Self-Determination  - Cultural Reclamation  - Economic development, and public health  - *Intergenerational knowledge, land-based practices, and resistance to colonial processed food systems* | - | - Growing Resilience Project: A community garden initiative supporting food sovereignty by teaching gardening skills, promoting intergenerational knowledge sharing, and encouraging community health.  - Revival of Buffalo Herds: Efforts to reintroduce to the land.  - Restoring Shoshone Ancestral Food Gathering: A community initiative reclaiming and sharing traditional gathering and cooking practices.  - Farmers Market & Food Initiatives: Led by Blue Mountain Associates, including the founding of the first tribal farmers market, support for home gardens, chicken coops, and producer development through the Wind River Food Sovereignty Development Project. |
| **“Ecological Calendars, Food Sovereignty, and Climate Adaptation in Standing Rock”** [**https://doi.org/10.1029/2022gh000621**](https://doi.org/10.1029/2022gh000621) | | | | | | |
| Ruelle et al.  (2022)^(46)^ | Community-based participatory qualitative study | Indigenous communities from the Standing Rock Nation, located in North Dakota and South Dakota, USA - Bullhead, Cannon Ball, Fort Yates, Kenel, Little Eagle, Porcupine, and Wakpala | “Indigenous food sovereignty relies on ecological knowledge of plants and animals, including knowledge related to their development and behavior through the seasons.” | – Ecological knowledge of plants and animals – Seasonal cues | -  Ecological calendars co-generated with Indigenous communities to document and apply seasonal ecological knowledge to support food sovereignty | - Use of seasonal ecological indicators (e.g., meadowlark song, first frost) to guide harvesting (e.g., buffalo berries) and planting  - Co-generation of community-specific ecological calendars with Elders and community members  - Revitalization of Indigenous names for moon cycles to track seasonal food activities  - Harvesting practices aligned with ecological cues, e.g., stopping red willow harvest after first thunder |
| **“Impacts of harvested species declines on Indigenous Peoples’ food sovereignty, well-being and ways of life: a case study of Anishinaabe perspectives and moose”** [**https://doi.org/10.5751/ES-12995-270130**](https://doi.org/10.5751/ES-12995-270130) | | | | | | |
| Priadka et al.  (2022)^(47)^  (Indigenous authors: Two Anishinaabekwe, three representatives from the lands departments of partner First Nations, and one non-Indigenous ally.) | Community-based participatory case study | Anishinaabe Nations in Ontario (Biigtigong Nishnaabeg, Magnetawan First Nation, Shawanaga First Nation) | “Food sovereignty through Indigenous-led governance will require a resurgence in policies that accept and include the perspectives and ways of life of Indigenous Peoples, particularly for decision-making related to food access and management *(Elliott et al. 2012, Rudolph and McLachlan 2013)*.” | - Self-governance  - Connection to land and traditional territory  - Culture, identity, spirituality  - Reciprocity and sharing food  - Autonomous monitoring and co-management | -  Autonomous community-based wildlife monitoring and co-management of traditional food sources (moose) | - Biigtigong Nishnaabeg holds an annual moose hunt camp where families gather to hunt and process moose.  - Intergenerational knowledge transmission: “Bringing the kids out and making sure that they learn all this stuff that we’ve never learned either as kids growing up. So, we’re also learning alongside them as well.”  - Community-based monitoring as a governance practice: Monitoring moose population and harvest by and for the community to support self-determination and sovereignty. |
| **“Indigenous food sovereignty through resurgent self-governance: centering Nuu-chah-nulth principles in sea otter management in Canada”** [**https://doi.org/10.5751/ES-13702-280212**](https://doi.org/10.5751/ES-13702-280212) | | | | | | |
| Popken et al. (2023)^(48)^  (Include Indigenous authors) | Qualitative analysis focused on Nuu-chah-nulth principles in sea otter management | Nuu-chah-nulth communities on Vancouver Island, Canada | “For Coté, food sovereignty stresses the need for collective relationships between humans and the natural world grounded in kinship, reciprocity, and respect, all of which emphasize how humans and the natural world care for and rely upon one another *(Coté 2022).”* *Kyle Whyte (2018)* “understands food sovereignty as rooted in relationships between human institutions and food systems, which are central to Indigenous communities’ “collective continuance,” or their “overall capacity to adapt to social and environmental changes, or resilience”.  “For Nuu-chah-nulth and many other Indigenous peoples, responsibility, obligation, and care of both human and non-human relationships is a core dynamic at the heart of food sovereignty *(Jurisdiction and Governance Mandate Working Group 1999, Griffin 2020)*.” | The study highlights three main principles central to Nuu-chah-nulth food sovereignty:  - hišukʔiš c̓awaak (Everything is one): Recognizing interconnectedness in the ecosystem and the importance of maintaining balance.  - ʔiisaak (Respect with caring): Respect for all beings, including sea otters, guided by responsible and sustainable harvesting.  - ʔuʔaałuk (Taking care of): A commitment to sustainability, with stewardship responsibilities toward sea otters and other species | - | - Traditional Sea Otter Management: The Nuu-chah-nulth historically practiced selective hunting to protect shellfish stocks, using their ecological knowledge to manage the otter population and ensure food availability for the community.  - Oceans Dialogue Forum: Nuu-chah-nulth and other Indigenous representatives organized a workshop to establish collaborative governance models for sea otter management, promoting food sovereignty through shared knowledge and traditional practices |
| **“Indigenous food sovereignty: Reclaiming food as sacred medicine in Aotearoa New Zealand and Peru”** [**https://dx.doi.org/10.20417/nzjecol.43.39**](https://dx.doi.org/10.20417/nzjecol.43.39) | | | | | | |
| Huambachano (2019)^(49)^  (Indigenous author) | Comparative study on Indigenous food sovereignty among Māori and Quechua peoples | Māori (Aotearoa New Zealand) and Quechua (Peru) | “While the conception of food sovereignty resonates with the aspirations of Indigenous peoples, Indigenous scholars point out that Indigenous food sovereignty transcends the legal and human ‘rights-based’ entrenched in the food sovereignty concept. Food sovereignty for Indigenous peoples emphasizes collective and cultural responsibilities that arise out of a network of descent and kinship relationships *(Coté 2016; Morrison 2016; Huambachano 2018).*” | - Sacred Kinship: Food is a gift from Pachamama (Andes) and Papatūānuku (New Zealand), symbolizing a spiritual bond with land  - Collective Self-Determination: Preservation of traditional food practices and TEK as acts of resilience  - Environmental Responsibility: Commitment to sustain biodiversity and cultural heritage through ecologically aligned food practices | - | - Māori communities: emphasize the use of the maramataka (lunar calendar) in gardening practices at Papatūānuku Marae. These gardens are planted using traditional practices, particularly for crops like kūmara, aligning with Māori spiritual and ecological principles. Hākari (ceremonial feasts) are conducted to celebrate the harvest, reinforcing kinship and cultural continuity.  - Quechua communities: practice sustainable agriculture rooted in their reverence for Pachamama. Rituals and ceremonies honor the land and ensure a balanced ecosystem, fostering resilience and maintaining their cultural ties to the landscape. |
| **“hishuk’ish tsawalk—Everything is one. Revitalizing place-based Indigenous food systems through the enactment of food sovereignty”** [**https://doi.org/10.5304/jafscd.2019.09A.003**](https://doi.org/10.5304/jafscd.2019.09A.003) | | | | | | |
| Coté (2019)^(50)^  (Indigenous author) | Keynote address adapted into a reflective case study | Tseshaht/Nuu-chah-nulth Nation, west coast of Vancouver Island, Canada | “Indigenous food sovereignty is defined within a restorative context that works to nurture individual and community health by repairing and fostering these healthy relationships.”  Indigenous food sovereignty is described within the framework of *hishuk’ish tsawalk-* everything is one - and emphasizes restoring health where relationships with land, water, and all life are treated as sacred and reciprocal responsibilities.  “Indigenizing this food sovereignty movement means moving it beyond a rights-based discourse to emphasize cultural responsibilities and relationships that indigenous peoples have with their environment.”  “All nations, including indigenous nations, have the right to define strategies and policies, and develop food systems and practices, that reflect their own cultural values around producing, consuming, and distributing food.” | - Sacred Sovereignty  -Participatory  - Self-Determination  -Policy  *(Morrison, 2011, pp. 100–101)*  - Responsibility, mutuality, kinship, and relationships *(WGIFS)*  The main principles central to Nuu-chah-nulth:  - hišukʔiš c̓awaak (Everything is one).  - ʔiisaak (Respect with caring).  - ʔuʔaałuk (Taking care of): | - | - Makah Whale Hunt: The Makah and Nuu-chah-nulth Nations’ whale hunts serve as powerful expressions of cultural identity and spiritual connection to the sea. These marine-based food systems reflect longstanding traditions of stewardship and place-based knowledge.  - Tseshaht Communal Fish Days: Community members gather early in the morning to fish and distribute salmon. These gatherings highlight intergenerational knowledge transmission, cultural continuity, and community bonding, reinforcing spiritual and ecological relationships with salmon.  - Tseshaht Garden Project: A garden planted on the grounds of a former residential school, now producing kale, squash, spinach, and more. The garden fosters community resilience, reclaims a space marked by trauma, and promotes healing relationships with land and food.  - Community harvesting of devil’s club, fern root (*ʔicmapt*), and salmonberries (*qawiisa*) in traditional territories (*ha-houłhee*), emphasizing place-based knowledge, family ties, and seasonal practices.  - Language and TEK Revitalization: Language lessons on the environment developed through videos with elders, connecting Nuu-chah-nulth language revitalization to traditional ecological knowledge and food systems. |
| **“Brief Research Commentary: The US Indigenous Food Sovereignty Movement’s Impact on Understandings of COVID-19 in Indian Country”**  [**https://doi.org/10.1111/cuag.12280**](https://doi.org/10.1111/cuag.12280) | | | | | | |
| Lewis  (2021)^(51)^  (Indigenous author) | Research commentary analyzing the intersection of Indigenous food sovereignty and the COVID-19 pandemic | US Indigenous communities, broadly | - | - “Regaining control of food is power” – *Self-determination*  - “Rematriation as a central tenet” – *cultural revitalization*  - “The linguistic and material linkages vital to the Indigenous food sovereignty movement” – *Language, knowledge, and cultural continuity* | - | Highlights COVID-19 adaptations by Native Nations to enhance food access and resilience during the pandemic. The Diné Nation’s commitment to a sustainable, nutritious, and resilient food system became critical during this period. The Diné Policy Institute’s Food Sovereignty Project engaged citizens through educational initiatives, while the Navajo Nation Council enacted food-related laws to support health and food sovereignty.  Many rejected standard federal government messaging on COVID-19, instead developing culturally grounded language, practices, and communication strategies. The Diné Nation named the virus “Dikos Ntsaaígíí-19,” and Cherokee Nation artists created public health flyers featuring Cherokee citizens and the Cherokee language, including syllabary, phonetic spellings, and English. |
| **“A critical race and class analysis of learning in the organic farming movement”** [**https://files.eric.ed.gov/fulltext/EJ1000191.pdf**](https://files.eric.ed.gov/fulltext/EJ1000191.pdf) | | | | | | |
| Etmanski, C.  (2012)^(52)^ | Theoretical, critical commentary with autobiographical reflection | Canada and North America, with a specific emphasis on Vancouver Island and the Gulf Islands in British Columbia. | “The movement of Indigenous food sovereignty therefore strengthens Indigenous people’s “ability to respond to our own needs for healthy, culturally adapted Indigenous foods” *(Indigenous Food Systems Network, n.d., section on Indigenous Food Sovereignty, para. 1)*.”  “Indigenous food sovereignty is fundamentally achieved by upholding our sacred responsibility to nurture healthy, interdependent relationships with the land, plants and animals that provide us with our food.”  “Reclaiming Indigenous food systems is an act of self-determination, empowerment, and resistance to ongoing racism and the effects of colonization.” | - Sacred o divine sovereignty  - Participatory  - Self-determination  - Policy  - Interdependent relationships with land, plants, and animals  - Empowerment and resistance to colonization | - | - Cultural harvesting strategies practiced at individual, family, community, and regional levels.  - Rainbow Chard Collective (British Columbia): A grassroots group advocates making food activism accessible to all.  - Reclaiming traditional food systems as part of broader acts of self-determination and resistance: “Across Canada and in various parts of the world, Indigenous peoples are mobilizing to promote, protect and, in some cases, reclaim pre-colonial practices related to food.” |
| **“An Intersectionality-Based Policy Analysis Examining the Complexities of Access to Wild Game and Fish for Urban Indigenous Women in Northwestern Ontario*”* doi: 10.3389/fcomm.2021.762083** | | | | | | |
| Philipps et al.  (2022)^(53)^ | Qualitative study using thematic analysis within an Intersectionality-Based Policy Analysis (IBPA) | Thunder Bay and Sioux Lookout (Northwestern Ontario) | “IFS respects struggles for self-determination within Canada, where food has been used as a tool of ongoing settler colonialization (Burnett et al., 2016; Martens et al., 2016). Policy is a pillar of IFS because oppressive land, water, food, economic, and environmental policies prohibit the land based practices necessary to enact food sovereignty *(Morrison, 2011; Morrison, 2020).”* | - Self-determination  - Policy  - Interdependency, respect, responsibility, and reciprocity  - Spirituality and cultural identity | - | Harvesting and sharing wild game; cultural food knowledge transmission within families;. |
| **“Indigenizing” Food Sovereignty. Revitalizing Indigenous Food Practices and Ecological Knowledges in Canada and the United States*”* doi:10.3390/h5030057** | | | | | | |
| Coté  (2016)^(54)^  (Indigenous author) | Theoretical/  Conceptual Analysis | Indigenous communities in Canada and the United States, with a focus on the Nuu-chah-nulth in British Columbia | “Indigenizing the food sovereignty movement means moving it beyond the rights-based discourse to emphasize cultural responsibilities and relationships that Indigenous peoples have with their environment. It also requires examining the efforts being made by Indigenous communities to restore these relationships through the revitalization of their Indigenous foods and ecological knowledge systems as they assert control over their own wellbeing.”  “While Indigenous communities are distinct, making it impossible to define food sovereignty in a way that reflects all of their realities, Morrison says they are united by eco-philosophical principles that have guided their interactions with the environment and the non-human world that has informed their food systems.” | General IFS principles:  - Sacred sovereignty: Food is a sacred gift from the Creator. - Participatory: Responsibility to nurture interdependent relationships with the ecosystem. - Self-determination: Freedom to meet community food needs independently. - Policy: Policies reconciling Indigenous food values with colonial laws.  - Eco-philosophy: Managing human behavior in relation to the land, contrasting with control over it.  Nuu-chah-nulth principles: • *Hishuk’ish tsawalk*: Everything is interconnected. • *Uu-a-thluk*: Taking care of lands and resources. • *Iisaak*: Respect for all life forms. | - | - Nuu-chah-nulth practices: Guided by *hishuk’ish tsawalk* (interconnectedness) and *iisaak* (respect), aiming to restore lands and develop traditional value-based policies.  - Sustainable fisheries management led by hereditary chiefs (Ha’wiih), combining ancestral laws with food and environmental policies. - Inuit practices: Traditional whaling holds cultural and spiritual significance, fostering community solidarity through shared practices that strengthen identity and heritage. |
| **“First foods as Indigenous food sovereignty: Country foods and breastfeeding practices in a Manitoban First Nations community*” 10.15353/cfs-rcea.v5i2.249*** | | | | | | |
| Cidro et al. (2018)^(55)^  (Include Indigenous authors) | Qualitative, community-based research as part of the Baby Teeth Talk (BTT) study | Norway House Cree Nation, northern Manitoba, Canada | “Indigenous food sovereignty (IFS) offers insights into the social, cultural, and environmental challenges of a deficient food system.”  “Indigenous Food Sovereignty (IFS) is a term that has only recently become conceptualized in research, but has long been practiced in First Nations communities (Morrison, 2011).”  “Indigenous food sovereignty argues for active participation in Indigenous food systems so that the mind, body, and spirit are nourished (Martens, 2015).” | - Cultural Continuity: Breastfeeding and country foods reinforce cultural practices and support infant health  The People’s Food Policy Program proposed four characteristics of IFS  - To return to the agreements of the treaties and reform and redistribute land  - To include the Indigenous concepts of harmony with nature in resource-based policy  - To address the socioeconomic determinants that are negatively affecting Indigenous health  - To rebuild the relationships between Indigenous peoples and other stakeholders *(PFPP, 2010).*  Morrison (2011) four principles of IFS:  - Sacred responsibility to the land  - Community self-determination  - Active participation in the food system  - Policy reform | - | - Highlights breastfeeding and country food introduction as early expressions of IFS, reinforcing cultural continuity and health. Includes country foods like fish and rabbit, shared through grandmothers’ teachings, as essential for infant development. |
| **“Nimíciwinán, nipimátisiwinán–“Our food is our way of life”: On-Reserve First Nation perspectives on community food security and sovereignty through oral history in Fisher River Cree Nation, Manitoba”** [**https://doi.org/10.15353/cfs-rcea.v6i2.218**](https://doi.org/10.15353/cfs-rcea.v6i2.218) | | | | | | |
| Shukla et al. (2023)^(56)^ | Participatory, place-based, collaborative case study | Fisher River Cree Nation (FRCN), Manitoba, Canada | “Indigenous food sovereignty (IFS) has been recognized as the strategic practice of Indigenous peoples to sustain traditional practices of harvesting, which include fishing, and hunting, and respecting the ‘sovereign rights and power of each distinct nation’ *(Morrison, 2011, p. 98)*”  “IFS recognizes and underscores the rights and uniqueness of Indigenous groups in defining and managing their own Indigenous food systems. ” | Morrison’s Framework: Sacred or divine sovereignty, participation,  self-determination,  legislation and policy  Community Control and Cultural Resilience: Emphasis on reclaiming food systems and addressing barriers such as environmental degradation and market dependency | - | Outdoor Education Program: FRCN’s outdoor education program engages youth in traditional food practices such as gathering, hunting, fishing, and wild harvesting for high school credit. The program is popular among youth and has a waiting list every year due to limited funding  Community Gardens and Self-Production: FRCN launched community garden projects in collaboration with University of Winnipeg to foster local food production and engage youth and elders in hands-on learning |
| **“Food sovereignty in Kalaallit Nunaat”** [**https://doi.org/10.1080/07409710.2024.2405795**](https://doi.org/10.1080/07409710.2024.2405795) | | | | | | |
| Hauptmann, A.L.  (2024)^(57)^  (Indigenous author) | Qualitative, narrative-based  Commentary | Kalaallit Nunaat (Greenland)  Inuit communities | Dawn Morrison (2011) argues that there is no universal definition of food sovereignty that reflects all the realities of Indigenous com-munities and that the concept of Indigenous food sovereignty describes strategies that support traditional food practices, which requires “a deepened cross-cultural understanding of the ways in which Indigenous knowledge, values, wisdom and practices can inform food-related action and policy reform” *(Morrison, 2011)*  Grey and Patel (2015) have defined the broader notion of food sovereignty as being “centrally, though not exclusively, about groups of people making their own decisions about the food system. and go on to argue that for the concept to be viable in Indigenous contexts, it must include a “continuation of anti-colonial struggles, even in post-colonial contexts” *(Grey and Patel, 2015)*  The concept of Indigenous food sovereignty allows a deeper analysis of the cultural dynamics and power inequalities in Kalaallit Nunaat. The Inuit Circumpolar Council of Alaska has developed an Inuit-specific concept of food sovereignty centered on Inuit values and world views (Inuit Circumpolar Council Alaska 2020). Inspired by this it becomes relevant to ask in the context of Kalaallit Nunaat, even when Inuit have the right to define our food system, which values and world view is represented in the system and why.  As Coté (2016) and Morrison (2011) point out, Indigenous food sovereignty emphasizes cultural responsibilities, relationships with the environment, and efforts to restore these relationships through revitalization of Indigenous foods. | - Responsibility to land  - Revitalization of Indigenous foods and food systems  - Cultural identity  - Resistance to colonial food systems  - Relationality | - | Revival of traditional practices such as fermenting eider eggs |
| **“Indigenous Food Sovereignty, Renewal and U.S. Settler Colonialism”** | | | | | | |
| Whyte  (2016)^(58)^  (Indigenous author) | Theoretical and conceptual essay examining Indigenous food sovereignty within the context of settler colonialism | Indigenous communities across North America with a focus on Anishinaabe (Great Lakes Region) | “Food sovereignty is a strategy of Indigenous resurgence that negotiates structures of settler colonialism that erase the ecological value of certain foods for Indigenous peoples.”  “Food sovereignty, for some North American Indigenous persons, is not really based on ideals of food self-sufficiency and cultural autonomy. Indeed, food sovereignty is a practical response to a particular structure of oppression that seeks to erase the ecologies that constitute Indigenous homelands.” | - Ecological value of food: Food serves as a “hub” linking cultural, social, ecological, and political dimensions. - Adaptive capacity: Ability to maintain food-related cultural practices and resilience amid settler colonialism. - Collective capacities: Community-based actions to cultivate, protect, and renew food practices.  - Self-determination - Resistance to settler colonialism: Recognizes settler policies as direct threats to Indigenous food systems. | - | Example of Anishinaabe-led wild rice restoration projects as a form of resistance to ecological degradation caused by settler activities, and as a means to reinforce community resilience and adaptive capacity in the face of colonial impacts. |
| **“Indigenous companion planting in the great churn: Three sisters in Kalapuya ilihi”** [**https://doi.org/10.1177/25148486221126618**](https://doi.org/10.1177/25148486221126618) | | | | | | |
| Klopotek et al.  (2022)^(59)^  (Indigenous authors) | Qualitative, narrative-based study (ethnographic) | Kalapuya ilihi (Western Oregon, USA) | “Specifically Indigenous food sovereignty, as [Coté (2016)](https://journals.sagepub.com/reader/content/18b02b4fbc7/10.1177/25148486221126618/format/epub/EPUB/xhtml/index.xhtml?hmac=1752631339-Omaq%2BRJxb65uWwWAlaJYIF0wSrdEhfEPdXRfwfV8Rf4%3D#bibr8-25148486221126618) describes it, “moves beyond a rights-based discourse by emphasizing the cultural responsibilities and relationships Indigenous peoples have with their environment and the efforts being made by Indigenous communities to restore these relationships through the revitalization of Indigenous foods and ecological knowledge systems as they assert control over their own foods and practices.” *(Coté, 2016)* | - Companion planting  - Indigenous mobility  - Relational community building  - Cultural responsibility | - | - Three Sisters agriculture in an effort to grow healthy, culturally relevant food and break from the cycle of reliance on unhealthy, overly processed foods and foodways imposed by colonial economies—in other words, to pursue Indigenous food sovereignty.  - Educational field trip: support the work of restoring Indigenous ecological knowledge and Kalapuya ecology itself. Connecting university and Indigenous community projects moves beyond territorial acknowledgments and towards restoring useable territory to tribes, even in places where outright ownership isn't restored. We work together to restore relationships of Indigenous peoples to their land. |
| **“Keeping ‘our’ land: property, agriculture and tensions between Indigenous and settler visions of food sovereignty in Canada”** [**https://doi.org/10.1080/03066150.2018.1439929**](https://doi.org/10.1080/03066150.2018.1439929) | | | | | | |
| Kepkiewicz & Dale  (2019)^(60)^ | Critical, theoretical paper | Canada | Indigenous food sovereignty frameworks, which underline Indigenous presence, Indigenous struggles and ongoing settler colonialism. As both a framework and a movement, Indigenous food sovereignty is based in four principles | Indigenous food sovereignty is based in four principles:  - The necessity of maintaining Indigenous relationships with land  - The ongoing work of Indigenous peoples in shaping healthy and culturally appropriate food systems  - The daily maintenance of Indigenous food systems by Indigenous peoples  - The need for Indigenous influence over policies at all jurisdictional levels *(Morrison 2008, 2011; see also Coté 2016; Martens 2015; Manson 2015; Martin and Amos2017).*  Indigenous food sovereignty builds on this view of land as relational, and is guided by four principles: sacredness, action-based participation, self-determination and policy *(Morrison 2011)* | - | - |
| **“Unsettling Settler Food Movements: Food Sovereignty and Decolonization in Canada”** [**https://doi.org/10.7202/1038478ar**](https://doi.org/10.7202/1038478ar) | | | | | | |
| Matties  (2016)^(61)^ | Theoretical analysis examining the intersections of settler colonialism, alternative food movements, and Indigenous food sovereignty | Indigenous communities across Canada | “Morrison points to Indigenous food sovereignty as a restorative framework for food systems transformation to which all people and cultures can relate.”  “IFS provides a critique of the often agriculture-centric discourses of global food sovereignty by adding that food provisioning practices such as hunting, fishing, gathering, and tending the environment must be engaged. For IFS “a ‘right to define agricultural policy’ is indistinguishable from a right to be Indigenous, in any substantive sense of the term.” *(Grey and Patel)* | 1.Food as Sacred  2.Focuses on food for people  3.Values food providers,  4.Localizes food systems,  5.Puts control locally,  6.Builds knowledge and skills,  7.Works with nature  Self-determination  Relationship with one another and the land, plants and animals | - | - |
| **“Return to Freedom: Anti-GMO Aloha ‘A¯ in a Activism on Molokai as an Expression of Place-based Food Sovereignty”**  [**http://dx.doi.org/10.1080/14747731.2014.957586**](http://dx.doi.org/10.1080/14747731.2014.957586) | | | | | | |
| Gupta, C.  (2014)^(62)^ | Qualitative case study | Native Hawaiian community in Molokai (Hawai‘i) | “In Canada for example, Indigenous peoples have self deﬁned Indigenous food sovereignty as a framework that includes the ‘freedom and ability to respond to our own needs for healthy, culturally adapted Indigenous foods. It represents the freedom and ability to make decisions over the amount and quantity of food we hunt, ﬁsh, gather, grow and eat’ *(Morrison, 2011, p. 100).*”  “In Hawai‘i, a conference on food sovereignty started annually on Hawai‘i Island in 2007, called Ho¯‘ea Ea (‘return to freedom’), described food sovereignty oriented around Native agricultural practices as ‘a spiritual, physical and cognitive pathway toward greater wellbeing and self-sufﬁciency’. In this way, food sovereignty is about securing the rights necessary to achieve food self-sufﬁciency.”  “Hawai‘i’s food sovereignty movement, which in turn is viewed by a growing number of Native Hawaiians as a pathway toward Indigenous sustainable self-determination.” | - Aloha ‘a¯in: Love for the land  - Kuleana: responsibility  - Self-determination | - | - Cultivation of kalo (taro), an Indigenous Hawaiian staple crop, is a sovereignty of autonomous livelihoods and self-determining collective personhoods, achieved through controlling one’s labor (inputs and outputs) *(Lasky, 2008).*  Meyer (2014) wrote recently, ‘There is a food sovereignty movement in Hawai‘i!’ She describes youth garden projects as one small link in a larger chain of gardens, communities, and creative ideas oriented around a Hawaiian view of the land, ‘a¯ina momona (fertile land), which are surfacing in Hawai‘i’s food sovereignty and Indigenous education movements.  Restoration of fishponds and subsistence practices (fishing, hunting, gathering) on Molokai.  Community organizing (e.g., Moms on a Mission, anti-GMO marches) promoting local control over food systems. |
| **“Kaˀtshatstʌ́sla: “Strength of belief and vision as a people”—A case study of Oneida resilience and corn”**  [**https://doi.org/10.5304/jafscd.2019.09B.015**](https://doi.org/10.5304/jafscd.2019.09B.015) | | | | | | |
| Stevens & Brewer  (2014)^(63)^  (1 Indigenous author) | Case study | Oneida nation of Wisconsin | ‘For the Onᴧyoteˀa·ká· in Wisconsin, food sovereignty was never about using food systems to exercise power; rather, like a number of Indigenous peoples, they have an engrained history of respecting the power within our food, which reflects an understanding that the universe is alive and therefore should be treated respectfully (Little Bear, 2000). And while the interconnected aspects of culture, heritage, politics, and place can make it difficult to define Indigenous food sovereignty for all Indigenous communities, the inherent power within o·nʌ́steˀ represents something that goes beyond the concept of food sovereignty. It is a tribal consciousness that is acted upon’ | Reciprocal relationship  Respecting the power within our food  Understanding that the universe is alive and therefore should be treated respectfully  Transference of knowledge across generations  Cultural and ceremonial responsibilities  The interconnected aspects of culture, heritage, politics, and place | - | At the husking  bee, the community comes together to harvest,  husk, and braid the cobs together into tall, beautiful  collections that are a physical representation of  years of resilience, imbued in the braids and  community working together to create the braids.  OCIFS is founded on a mission to help families with dietary and food needs by housing a community initiative, which incorporates traditional foods to help create as well as reestablish a local economy that provides jobs and promotes and encourages long-term solutions to farm and nutrition issues on the Oneida Reservation.  Bringing together eight families, securing funding  through grants, utilizing very old but still intact  knowledge systems in Tsyunhehkw^ staff members,  and purchasing seeds from the Onondaga  Nation in New York |
| **“Seeking Indigenous food sovereignty: origins of and responses to the food crisis in northern Manitoba, Canada”** [**https://doi.org/10.1080/13549839.2012.754741**](https://doi.org/10.1080/13549839.2012.754741) | | | | | | |
| Rudolph & McLachlan  (2013)^(64)^ | Qualitative study | Grand Rapids and Misipawistik Cree Nation (MCN), northern Manitoba, Canada | “Indigenous food sovereignty acknowledges the unique offerings of Indigenous communities to alternative system. These include traditional knowledge, innovations, and land-use practices that serve as living viable alternatives to globalisation *(Carino 2006)* and as examples of community-based control that results in sustainable food production and genuine food security *(Wiebe and Wipf 2011)*.”  “Indigenous food sovereignty framework explicitly connects the health of food with the health of the land and identiﬁes a history of social injustice as having radically reduced Indigenous food sovereignty in colonised nations *(PFPP 2010).*” | Morrison (2011) indicates that Indigenous food sovereignty comprises four central tenets: that the sacred responsibilities and relationships with the land supersede any colonial laws or policies; that the ability to define and respond to food-related needs should be self-determined; that these goals should be achieved through continued participation in these food systems at all scales of organisation; and that these ends can ultimately only be achieved through policy and legislative reform *(Morrison 2011, p. 100).*  Traditional knowledge, innovations, and land-use  “… Food security – an essential component of Indigenous food sovereignty and an opportunity that was and that continues to be denied.” | - | Revival of country foods and treaty rights  *“Procuring country foods had richer meanings beyond food security, as it affirmed treaty rights and represented an opportunity to engage in cultural and spiritual connections with the land.”*  Gardening as cultural and community practice  *“The power of community gardens […] has less to do with food and much more to do with building community, sharing knowledge between youth and Elders, generating a sense of pride and empowerment…”*  Traditional food sharing  *“Traditional share-culture is still a fundamental aspect of modern-day Cree life. Continuing a free, community-operated food distribution system, particularly for Elders and those in need, was thus important…”*  Nation-to-nation food exchange  *“Trading relationships with potatoes, corn, and other vegetables being exchanged for country foods including moose and ﬁsh.”* |
| **“Farmers, foodies and First Nations: getting to food sovereignty in Canada”** [**http://dx.doi.org/10.1080/03066150.2013.876623**](http://dx.doi.org/10.1080/03066150.2013.876623) | | | | | | |
| Desmarais & Wittman  (2014)^(65)^ | Qualitative, conceptual analysis | Indigenous communities in Canada | “Indigenous food sovereignty describes, rather than deﬁnes, the present day strategies that enable and support the ability of Indigenous communities to sustain traditional hunting, ﬁshing, gathering, farming and distribution practices, the way we have done for thousands of years prior to contact with the ﬁrst European settlers … We have rejected a formal universal deﬁnition of sovereignty in favour of one that respects the sovereign rights and power of each distinct nation to identify the characteristics of our cultures and what it means to be Indigenous *(Morrison, 2011)*.”  “Indigenous food sovereignty activists stress the importance of decolonization, self-determination and the inclusion of fishing, hunting and gathering as key elements of a food sovereignty approach to sustainable food systems in Canada, and highlight the complexity of issues of sovereignty, authority, individual and collective rights, equity, culture and (re)distribution of land and other resources *(Morrison, 2006, 2011).*”  “Concept of indigenous food sovereignty, a framework that explicitly recognizes the social, cultural and economic relationships that underlie inter-community food sharing and trading as a mechanism for indigenous health and well-being.” | - Decolonization,  - Self-determination  - The inclusion of ﬁshing, hunting and gathering  - Authority,  - Individual and collective rights,  - Equity,  - Culture and (re)distribution of land and other resources  - Inter-community food sharing and trading  Food is sacred, these discussions have emphasized the importance of Participation at individual, family, community, and regional levels. Self-determination refers to the ‘freedom and ability to respond to our own needs for healthy, culturally-adapted indigenous foods. It represents the freedom and ability to make decisions over the amount and quality of food we hunt, ﬁsh, gather, grow and eat’*(Morrison 2011, 100).* Finally, Legislation and policy reform attempts to ‘reconcile Indigenous food and cultural values with colonial laws, policies and mainstream economic activities’. This principle has resulted in signiﬁcant mobilization around policy reform in forestry, ﬁsheries and health programming. | - | Nuu-chah-nulth Fisheries Case (British Columbia):  A decade-long court battle finalized in 2009, affirming the Nation’s right to implement fishing and harvesting strategies based on its own cultural, economic, and ecological values.  Protests Against Open-Pen Salmon Farming: Indigenous groups joined environmental activists to oppose farmed Atlantic salmon operations that threaten wild salmon. |
| **“The dispute over wild rice: an investigation of treaty agreements and Ojibwe food sovereignty”** [**https://doi.org/10.1007/s10460-016-9703-6**](https://doi.org/10.1007/s10460-016-9703-6) | | | | | | |
| Raster & Gish Hill  (2016)^(66)^ | Case study | Ojibwe communities in the U.S. midwest | “While most Indigenous people today strive to attain food sovereignty, Indigenous foodways historically were not expressly focused on the right to attain food or control a production system. Rather, these food systems have primarily been about maintaining culturally, ecologically, and spiritually appropriate relationships with the plants and animals that provide food. Such a food system emphasizes reciprocal relationships with the entities on the landscape rather than asserting rights over particular resources as a means of controlling production and access.”  “We subsume Whyte’s notion of food justice in our definition of Indigenous food sovereignty on the premise that the relationships that Native peoples have developed over centuries of living within a particular landscape are vital to their ability to access food in ways that are culturally meaningful.” | - Food justice and sovereignty as rights grounded in cultural self-determination  - Self-determination over food systems and landscape management  - Reciprocal relationships with the land, plants, and animals that provide food  - Culturally, ecologically, and spiritually appropriate food systems  - Connection to cultural identity and tradition | - | Ongoing Wild Rice Harvesting and Protection against non-Indigenous and commercial threats. Advocacy and legal defense to maintain traditional food rights.  White Pine Treaty as recognition of Ojibwe food sovereignty: White Pine Treaty of 1837 specifically recognized Ojibwe food sovereignty and the people’s right to manage and maintain important relationships with their food resources.  ‘Ojibwe leaders recognized that access to their homelands and traditional resources was critical not only to their survival as a people but also to their political autonomy; thus, treaty rights that protected the Ojibwe’s traditional livelihoods were essential for helping them to navigate these changes’  Resistance to Genetic Modification : Ojibwe leaders opposed genetic research on wild rice, viewing it as a violation of their reciprocal relationship with the plant and ecosystem.  “To genetically engineer wild rice would be disrespectful to the First People who inhabited this land… It would be morally wrong.” Chairman Goggleye Jr., Leech Lake Band of Ojibwe. |
| **“Enacting food sovereignty in Aotearoa New Zealand and Peru: revitalizing Indigenous knowledge, food practices and ecological”** [**https://doi.org/10.1080/21683565.2018.1468380**](https://doi.org/10.1080/21683565.2018.1468380) | | | | | | |
| Huambachano, M.  (2018)^(67)^  (Indigenous author) | Comparative case study | Māori communities in Aotearoa and Quechua communities in Peru | Indigenous food sovereignty underscores the revitalization of Indigenous food systems as a tool for reclaiming their traditional food practices and principles and moving beyond colonial approaches food security *(Declaration of Nyéléni 2007; Huambachano 2015; LaDuke 2017; La Via Campesina 2011; Nelson 2008).*  “Quechua’s and Māori’s food sovereignty frameworks enact both food security as a legal and human right and food sovereignty as the right of peoples to *healthy and culturally appropriate food*. The latter reclaims the spiritual, cultural, and physical relationships-based approach that Indigenous people have with the land and all their living relations (Declaration of Nyéléni 2007; De Schutter 2009).” | - Self-determination  - Revitalization of their food systems  For Quechua communities (Peru):  -Ayni (reciprocity)  - Ayllu (collectiveness/self-governance)  - Yanantin and Mansitin (duality/complementarity)  - Chaninchay (solidarity)  *For Māori communities (Aotearoa):*  -Kaitiakitanga (guardianship)  - Whakapapa (kinship)  - Rāhui (spiritual environmental restriction)  -Wairuatanga (spirituality)  - Koha (reciprocity/gift)  - Tikanga (ethics) | -  No standardized tool is used. The study applies an Indigenous knowledge-based analytical framework, using Traditional Ecological Knowledge (TEK), cultural values (e.g., ayni, kaitiakitanga, koha), and narratives from Quechua and Māori participants to examine and articulate Indigenous food sovereignty frameworks. | The case studies demonstrate how Quechua and Māori communities enact Indigenous Food Sovereignty through daily practices:  *Quechua*: Bartering systems and chuño (dried potatoes) as food preservation and non-market food access strategies; ayni as a communal farming and reciprocal labour practice.  *Māori*: Use of rāhui (spiritual restrictions) to protect food sources; planting and harvesting guided by karakia (prayers); the gifting and return of land as an act of koha (reciprocity) following the Mount Tarawera eruption. |
| **“An appreciative inquiry and inventory of Indigenous food sovereignty initiatives within the western U.S.”** [**https://doi.org/10.5304/jafscd.2022.112.016**](https://doi.org/10.5304/jafscd.2022.112.016) | | | | | | |
| Babcock & Budowle  (2021)^(68)^ | Systematic inventory | Indigenous communities in western U.S. | “Indigenous food sovereignty is informed by—and is a framework and movement that supports—all the various means through which Indigenous people are revitalizing and reclaiming their traditional foodways.”  “Indigenous food sovereignty (IFS), which is the “ability of an [i]indigenous nation or community to control its own food system and food-producing resources free of control or limitations put on it by an outside power (such as a settler/colonizer government)” *(Indian Education Division, n.d., para. 1).*”  “IFS requires human responsibility tot he natural world, ensuring healthy food and ecosystems to support mutually beneficial relationships *(Hoover, 2017).*”  “IFS aims “to honor, value, and protect traditional food practices and networks in the face of ongoing pressures of [colonialism]” *(Desmarais & Wittman, 2014, p. 1165).*” | (1) sacred or divine sovereignty restoring land-based relationships; (2) active participation to maintain land, soil, water, air, plants, and animals; (3) self-determination to maintain freedom from colonial systems; (4) culturally appropriate legislation and policy *(Morrison, 2011).*  IFS often highlights history, identity, land reform and redistribution, environmental restoration, and social determinants of health *(People’s Food Policy Project, 2011; Robin, 2019).*  *-* Relationality, responsibility, reciprocity, and respect *(Coté, 2016; Hoover, 2017; Kimmerer, 2013; Morrison, 2011; Robin, 2019).*  - Kinship and ecological family sharing ancestry  - Autonomy, self-sufficiency, and self-determination | -  Open-access inventory created as a support resource | WahZahZee Osage Nation’s Bird Creek Farm (Oklahoma): *“Provides produce and other food to the Elder Nutrition Program, Head Start, and community cultural events.”*  Standing Rock Sioux Nation’s Native Garden Project (South Dakota): *“Has an Elders Advisory Board that plans youth trips for learning stories and Lakota food-gathering practices.”*  Cultural Conservancy (California Bay Area): “Integrates youth and Elders into all of its work, serving not only living generations, but also our ancestors and descendants.”  Sogorea Te’ Land Trust (California): *Acquires land to restore Native American foodways and create a sacred space for ceremony.*  Zuni Youth Enrichment Program : *“Provides summer camp experiences to learn traditional food knowledge and grow empowerment.”*  Teca WaWokiye Cokata: *“Supports hunting by teaching skills like bow-and-arrow-making and hide preparation.”*  Sierra Seeds: *Cultivates intimacy with the earth and ancestral food traditions through medicinal storytelling on seed songs and seed rematriation.* |
| **“Tracing the terrain of Indigenous food *sovereignties”*** [***https://doi.org/10.1080/03066150.2017.1324423***](https://doi.org/10.1080/03066150.2017.1324423) | | | | | | |
| Daigle, M.  (2019)^(69)^ | Qualitative, ethnographic approach; analytical and theoretical study | Anishinaabe communities in Treaty #3 territory (northwestern Ontario) | “Indigenous food sovereignty is ultimately achieved by upholding our long-standing sacred responsibilities to nurture healthy, interdependent relationships with the land, plants and animals that provide us with our food. *(Morrison 2011, 100)*”  “Grey and Patel argue that Indigenous food sovereignty efforts are, at once, forms of resistance against colonial-capi-talist legacies and continuities, and part of a more general political, legal and economic resurgence (Grey and Patel 2015). In this way, Grey and Patel (2015) characterize sovereignty in Indigenous Food sovereignty as both a resistance against the inter-workings of settler colonialism and neoliberal capitalism, and as a resurgence of Indigenous forms of authority and autonomy.” | - Sacred responsibilities and interdependence with land, plants, and animals  - Resistance and resurgence  - Self-determination and Indigenous governance  - Relationality with non-human kin  - Kinship and intergenerational knowledge transmission | - | Fall harvest gathering; intergenerational trapping workshops (e.g., Jones family); youth summer camps on food harvesting grounds; Lac La Croix fishing across the Canada–US border; reclaiming residential school sites and ancestral islands for communal food harvesting. |
| **“Food sovereignty, justice and indigenous peoples: an essay on settler colonialism and collective continuance”** [**https://doi.org/10.1093/oxfordhb/9780199372263.013.34**](https://doi.org/10.1093/oxfordhb/9780199372263.013.34) | | | | | | |
| Whyte, K.P.  (2017)^(70)^ | Essay |  | "Violating indigenous food sovereignty is a strategy of settler colonial domination that erases Indigenous capacities for exercising collective self-determination."  "Food sovereignty can be interpreted as based on particular qualities of relationships that promote a society’s overall adaptive capacity." | Collective self-determination  Responsibilities of care and reciprocity  Relationships | - | Anishinaabe: Wild rice harvesting as tied to treaty rights and cultural identity  Treaty Tribes (Pacific Northwest): Salmon fishing and habitat protection as essential to food sovereignty  Nuu-chah-nulth and other Northwest Coast nations: Potlatch ceremonies for redistributing salmon and reinforcing ecological stewardship  Karuk Nation: Cultural burning to maintain ecosystems for acorns, berries, and other traditional foods. Hunting and foraging as part of subsistence, ceremony, and barter systems. Resistance to criminalization of traditional food practices |
| **““You Can't Say You're Sovereign if You Can't Feed Yourself”: Defining and Enacting Food Sovereignty in American Indian Community Gardening”** [***https://doi.org/10.17953/aicrj.41.3.hoover***](https://doi.org/10.17953/aicrj.41.3.hoover) | | | | | | |
| Hoover, E.  (2017)^(71)^ | Qualitative descriptive study | Indigenous communities across the United States | “Put simply, indigenous food sovereignty “refers to a re-connection to land-based food and political systems,” *(Martens et al.)* and seeks to uphold “sacred responsibilities to nurture relationships with our land, culture, spirituality, and future generations.” *(Morrison, 2011)*  “Secwepemc scholar Dawn Morrison describes indigenous food sovereignty as a framework for exploring the right conditions for “reclaiming the social, political, and personal health we once experienced prior to colonization. But the framework itself does not resolve where the responsibility for it lies.”  “Karla Rae Rudolph and Stephane McLachlan describe, “An indigenous food sovereignty framework explicitly connects the health of food with the health of the land and identifies a history of social injustice as having radically reduced indigenous food sovereignty in colonized nations.”  “The concept of indigenous food sovereignty is not just focused on rights to land and food and the ability to control a production system, but also responsibilities to them, which encompasses culturally, ecologically, and spiritually appropriate relationships with elements of those systems.” | - They seek to stress the importance of communal culture, decolonization, and self-determination, as well as the inclusion of fishing, hunting, and gathering—rather than just agriculture—as key elements of a food sovereignty approach.  - Reciprocal relationships  - Responsibilities to land, and culturally, ecologically, and spiritually appropriate relationships with elements of food systems.  - Education and transmission of knowledge  (1) The right to food is sacred and must be upheld through sacred responsibilities to land, plants, and animals; (2) Daily participation at all levels (individual to regional) is fundamental; (3) Self-determination to make decisions over the amount and quality of food hunted, fished, gathered, grown, and eaten; (4) Legislation and policy must support Indigenous food and cultural values in contrast to colonial laws and systems. *(Dawn Morrison and the Working Group on Indigenous Food Sovereignty’s)* | - | Tohono O’odham Bahidaj Camp: Youth and community members harvest saguaro cactus fruit in a four-day camp that includes ceremony and story-sharing.  Mvskoke Food Sovereignty Initiative (Oklahoma): Partners with a Euchee language immersion school to garden in the Euchee language, linking food, education, and cultural revitalization.  Kanenhi:io Ionkwaienthon:hakie (Mohawk, Akwesasne): Community members are supported to access land and tools to grow their own food, emphasizing autonomy and seed saving.  White Earth Land Recovery Project (Ojibwe, Minnesota): Uses gardening and wild plant harvesting to teach cultural history, food relationships, and language.  Cheyenne River Youth Project (Lakota): Operates a community garden to combat diabetes and reconnect youth with traditional foodways.  Mobile Farmers Market aims to reconnect trade relationships and support community-to-community food sovereignty. |

**References:**

1.	Abdul M, Ingabire A, Lam CYN, Bennett B, Menzel K, MacKenzie-Shalders K, et al. Indigenous food sovereignty assessment-A systematic literature review. Nutrition & dietetics : the journal of the Dietitians Association of Australia. 2024;81(1):12-27.

2.	Gutierrez BV, Kaloostian D, Redvers N. Elements of Successful Food Sovereignty Interventions within Indigenous Communities in the United States and Canada: a Systematic Review. Current developments in nutrition. 2023;7(9):101973.

3.	Maudrie TL, Colón-Ramos U, Harper KM, Jock BW, Gittelsohn J. A Scoping Review of the Use of Indigenous Food Sovereignty Principles for Intervention and Future Directions. Current developments in nutrition. 2021;5(7):nzab093.

4.	Heeringa KM, Huntington O, Woods B, Chapin FS, Hum RE, Brinkman TJ, et al. A holistic definition of healthy traditional harvest practices for rural Indigenous communities in Interior Alaska. Journal of Agriculture Food Systems and Community Development. 2019;9:115-29.

5.	Datta R. Community-led food resilience:A decolonizing autographic learning from an Inuit community. Global Food Security-Agriculture Policy Economics and Environment. 2021;30.

6.	Affonso H, Fraser JA, Nepomuceno I, Torres M, Medeiros M. Exploring food sovereignty among Amazonian peoples: Brazil's national school feeding programme in Oriximiná, Pará state. Journal of Peasant Studies. 2024.

7.	Brant S, Williams K, Andrews J, Hammelman C, Levkoe CZ. Indigenous food systems and food sovereignty: A collaborative conversation from the American Association of Geographers 2022 Annual Meeting. Journal of Agriculture Food Systems and Community Development. 2023;12(3):141-54.

8.	Ray L, Burnett K, Cameron A, Joseph S, LeBlanc J, Parker B, et al. Examining Indigenous food sovereignty as a conceptual framework for health in two urban communities in Northern Ontario, Canada. Global health promotion. 2019;26(3_suppl):54-63.

9.	Wires KN, LaRose J. Sogorea Te' Land Trust empowers indigenous food sovereignty in the San Francisco Bay Area COMMENT. Journal of Agriculture Food Systems and Community Development. 2019;9:31-4.

10.	Maunakea AK, Juarez R, Maunakea-Forth JK. The Mauli Ola Study: A Unique Academic-Community Partnership With MA'O Organic Farms to Understand and Address Health Inequities Among Native Hawaiians and Other Pacific Islanders in Hawai'i. Health promotion practice. 2023;24(6):1087-90.

11.	Blue Bird Jernigan V, Maudrie TL, Nikolaus CJ, Benally T, Johnson S, Teague T, et al. Food sovereignty indicators for Indigenous community capacity building and health. Frontiers in Sustainable Food Systems. 2021;5:704750.

12.	Weiler AM, Hergesheimer C, Brisbois B, Wittman H, Yassi A, Spiegel JM. Food sovereignty, food security and health equity: a meta-narrative mapping exercise. Health policy and planning. 2015;30(8):1078-92.

13.	Paul KL, Ruppel KT, Young MM, Caplins L, Ramaker JF, Carter CJ, et al. Blackfeet innovation pathways to food sovereignty: sustainability through indigenous-led research partnerships. Frontiers in Sustainable Food Systems. 2024;8.

14.	Cidro J, Martens T, Guilbault L. Traditional Indigenous food upskilling as a pathway to urban Indigenous food sovereignty. Indigenous perspectives on education for well-being in Canada. 2016;41.

15.	Martens T, Cidro J, Hart MA, McLachlan S. Understanding Indigenous food sovereignty through an Indigenous research paradigm. Journal of Indigenous Social Development. 2016;5(1).

16.	Morrison D. Indigenous food sovereignty: A model for social learning. Food sovereignty in Canada: Creating just and sustainable food systems. 2011;97:113.

17.	Stiegman M, Pictou S. We Story the Land: Exploring Mi'kmaq food sovereignty, Indigenous law and treaty relations. Journal of Peasant Studies. 2024;51(2):294-317.

18.	Cidro J, Adekunle B, Peters E, Martens T. Beyond food security: Understanding access to cultural food for urban Indigenous people in Winnipeg as Indigenous food sovereignty. Canadian Journal of Urban Research. 2015;24(1):24-43.

19.	Rowe S, Brady C, Sarang R, Wiipongwii T, Leu M, Jennings L, et al. Improving Indigenous Food Sovereignty through sustainable food production: a narrative review. Frontiers in Sustainable Food Systems. 2024;8.

20.	Sumner J, McMurtry JJ, Tarhan D. Growing community sustenance: The social economy as a route to indigenous food sovereignty. Canadian Journal of Nonprofit and Social Economy Research. 2023;14(S1):16 pp- pp.

21.	Robin T. Our hands at work: Indigenous food sovereignty in Western Canada. Journal of Agriculture, Food Systems, and Community Development. 2019;9(B):85-99.

22.	Poirier B, Soares G, Neufeld HT, Hedges J, Sethi S, Jamieson L. Conceptualising the relationships between food sovereignty, food security, and oral health among global Indigenous Communities: A scoping review. Public Health Nutrition. 2024:1-24.

23.	Nguyen CJ, Wilbur RE, Henderson A, Sowerwine J, Mucioki M, Sarna-Wojcicki D, et al. Framing an Indigenous Food Sovereignty Research Agenda. Health promotion practice. 2023;24(6):1117-23.

24.	Ferguson CE, Green KM, Swanson SS. Indigenous food sovereignty is constrained by “time imperialism”. Geoforum. 2022;133:20-31.

25.	McEachern LW, Yessis J, Zupko B, Yovanovich J, Valaitis R, Hanning RM. Learning circles: an adaptive strategy to support food sovereignty among First Nations communities in Canada. Applied physiology, nutrition, and metabolism = Physiologie appliquee, nutrition et metabolisme. 2022;47(8):813-25.

26.	Levkoe CZ, McLaughlin J, Strutt C. Mobilizing networks and relationships through indigenous food sovereignty: the indigenous food circle’s response to the COVID-19 pandemic in Northwestern Ontario. Frontiers in Communication. 2021;6:672458.

27.	Blanchet R, Willows N, Johnson S, Batal M. Enhancing cultural food security among the Syilx Okanagan adults with the reintroduction of Okanagan sockeye salmon. Applied physiology, nutrition, and metabolism = Physiologie appliquee, nutrition et metabolisme. 2022;47(2):124-33.

28.	Young L, Shukla S, Wilson T. Indigenous values and perspectives for strengthening food security and sovereignty: learning from a community-based case study of <i>Misko-ziibiing</i> (Bloodvein River First Nation), Manitoba, Canada. Frontiers in Sustainable Food Systems. 2024;8.

29.	Miltenburg E, Neufeld HT, Anderson K. Relationality, Responsibility and Reciprocity: Cultivating Indigenous Food Sovereignty within Urban Environments. Nutrients. 2022;14(9).

30.	Poirier B, Neufeld HT. "We Need to Live off the Land": An Exploration and Conceptualization of Community-Based Indigenous Food Sovereignty Experiences and Practices. International journal of environmental research and public health. 2023;20(5).

31.	Kamal AG, Linklater R, Thompson S, Dipple J. A recipe for change: Reclamation of Indigenous food sovereignty in O-Pipon-Na-Piwin Cree Nation for decolonization, resource sharing, and cultural restoration. The Politics of Food Sovereignty: Routledge; 2018. p. 137-54.

32.	Miltenburg E, Neufeld HT, Perchak S, Skene D. "Where Creator Has My Feet, There I Will Be Responsible": Place-Making in Urban Environments through Indigenous Food Sovereignty Initiatives. International journal of environmental research and public health. 2023;20(11).

33.	MacNeill T. Indigenous food sovereignty in a captured state: the Garifuna in Honduras. Third World Quarterly. 2020;41(9):1537-55.

34.	Sowerwine J, Sarna-Wojcicki D, Mucioki M, Hillman L, Lake FK, Friedman E. Enhancing Indigenous food sovereignty: A five-year collaborative tribal-university research and extension project in California and Oregon. Journal of Agriculture Food Systems and Community Development. 2019;9:167-90.

35.	Hartmann K. Food Sovereignty in Indigenous Communities: Extension Programs for Health, Culture, and Resilience. Journal of Extension. 2023;61(3).

36.	Jernigan VBB, Nguyen CJ, Maudrie TL, Demientieff LX, Black JC, Mortenson R, et al. Food Sovereignty and Health: A Conceptual Framework to Advance Research and Practice. Health promotion practice. 2023;24(6):1070-4.

37.	Alabi BO, Robin T. Food insecurities and dependencies: Indigenous food responses to COVID-19. AlterNative (Auckland, NZ : 2005). 2023;19(1):204-10.

38.	Dower B, Gaddis J. Relative to the landscape: Producer cooperatives in native food sovereignty initiatives. Journal of Co-Operative Organization and Management. 2021;9(2).

39.	Maudrie TL, Nguyen CJ, Wilbur RE, Mucioki M, Clyma KR, Ferguson GL, et al. Food Security and Food Sovereignty: The Difference Between Surviving and Thriving. Health promotion practice. 2023;24(6):1075-9.

40.	Blanchet R, Batal M, Johnson-Down L, Johnson S, Willows N. An Indigenous food sovereignty initiative is positively associated with well-being and cultural connectedness in a survey of Syilx Okanagan adults in British Columbia, Canada. BMC public health. 2021;21(1):1405.

41.	Jonasson ME, Spiegel SJ, Thomas S, Yassi A, Wittman H, Takaro T, et al. Oil pipelines and food sovereignty: threat to health equity for Indigenous communities. Journal of public health policy. 2019;40(4):504-17.

42.	Ritchie I. Food Sovereignty in Whaingaroa: Perspectives of Food Providers in a Small, Coastal New Zealand Township. Anthropological Forum. 2016;26(3):289-300.

43.	Farfan JCC, Medina DFR, Ninco YA, Marulanda SC, Torres DA. Food sovereignty and autonomy for indigenous health as resistance to food globalization: Scoping review. Global Food Security. 2023;37:100682.

44.	Grey S, Newman L. Beyond culinary colonialism: indigenous food sovereignty, liberal multiculturalism, and the control of gastronomic capital. Agriculture and Human Values. 2018;35(3):717-30.

45.	Arthur ML, Porter CM. Restorying Northern Arapaho food sovereignty. J Agric Food Syst Community Dev. 2019;9(B Suppl 2):69-84.

46.	Ruelle ML, Skye AJ, Collins E, Kassam KS. Ecological Calendars, Food Sovereignty, and Climate Adaptation in Standing Rock. GeoHealth. 2022;6(12):e2022GH000621.

47.	Priadka P, Moses B, Kozmik C, Kell S, Popp JN. Impacts of harvested species declines on Indigenous Peoples’ food sovereignty, well-being and ways of life: a case study of Anishinaabe perspectives and moose. Ecology and Society. 2022;27(1).

48.	Popken LR, Griffin PJ, Cote C, Angel E. Indigenous food sovereignty through resurgent self-governance: centering Nuu-chah-nulth principles in sea otter management in Canada. Ecology and Society. 2023;28(2).

49.	Huambachano MA. Indigenous food sovereignty: Reclaiming food as sacred medicine in Aotearoa New Zealand and Peru. New Zealand Journal of Ecology. 2019;43(3).

50.	Coté C. <i>hishuk</i>'<i>ish tsawalk</i>- Everything is one. Revitalizing place-based Indigenous food systems through the enactment of food sovereignty. Journal of Agriculture Food Systems and Community Development. 2019;9:37-48.

51.	Lewis C. Brief Research Commentary: The US Indigenous Food Sovereignty Movement's Impact on Understandings of COVID-19 in Indian Country. Culture Agriculture Food and Environment. 2021;43(2):107-13.

52.	Etmanski C. A critical race and class analysis of learning in the organic farming movement. Australian Journal of Adult Learning. 2012;52(3):484-506.

53.	Phillipps B, Skinner K, Parker B, Neufeld HT. An Intersectionality-Based Policy Analysis Examining the Complexities of Access to Wild Game and Fish for Urban Indigenous Women in Northwestern Ontario. Frontiers in Communication. 2022;6.

54.	Coté C. “Indigenizing” food sovereignty. Revitalizing Indigenous food practices and ecological knowledges in Canada and the United States. Humanities. 2016;5(3):57.

55.	Cidro J, Martens TR, Zahayko L, Lawrence HP. First foods as Indigenous food sovereignty: Country foods and breastfeeding practices in a Manitoban First Nations community. 2018.

56.	Shukla S, Alfaro J, Cochrane C, Garson C, Mason G, Dyck J, et al. Nimíciwinán, nipimátisiwinán–“Our food is our way of life”: On-Reserve First Nation perspectives on community food security and sovereignty through oral history in Fisher River Cree Nation, Manitoba. Canadian Food Studies/La Revue canadienne des études sur l'alimentation. 2019;6(2):73-100.

57.	Hauptmann AL. Food sovereignty in Kalaallit Nunaat. Food and Foodways. 2024.

58.	Whyte KP. Indigenous food sovereignty, renewal, and US settler colonialism. The Routledge handbook of food ethics: Routledge; 2016. p. 354-65.

59.	Klopotek B, Claybrook T, Scott J. Indigenous companion planting in the great churn: Three sisters in Kalapuya ilihi. Environment and Planning E-Nature and Space. 2023;6(3):1889-904.

60.	Kepkiewicz L, Dale B. Keeping 'our' land: property, agriculture and tensions between Indigenous and settler visions of food sovereignty in Canada. Journal of Peasant Studies. 2019;46(5):983-1002.

61.	Matties Z. Unsettling settler food movements: Food sovereignty and decolonization in Canada. Cuizine. 2016;7(2).

62.	Gupta C. Return to Freedom: Anti-GMO Aloha 'Aina Activism on Molokai as an Expression of Place-based Food Sovereignty. Globalizations. 2015;12(4):529-44.

63.	Stevens LL, Brewer JP. Ka'tshatstsla: "Strength of belief and vision as a people"-A case study of Oneida resilience and corn. Journal of Agriculture Food Systems and Community Development. 2019;9:225-38.

64.	Rudolph KR, McLachlan SM. Seeking Indigenous food sovereignty: origins of and responses to the food crisis in northern Manitoba, Canada. Local Environment. 2013;18(9):1079-98.

65.	Desmarais AA, Wittman H. Farmers, foodies and First Nations: getting to food sovereignty in Canada. Journal of Peasant Studies. 2014;41(6):1153-73.

66.	Raster A, Hill CG. The dispute over wild rice: an investigation of treaty agreements and Ojibwe food sovereignty. Agriculture and Human Values. 2017;34(2):267-81.

67.	Huambachano M. Enacting food sovereignty in Aotearoa New Zealand and Peru: Revitalizing Indigenous knowledge, food practices and ecological philosophies. Agroecology and sustainable food systems. 2018;42(9):1003-28.

68.	Babcock A, Budowle R. An appreciative inquiry and inventory of Indigenous food sovereignty initiatives within the western U.S. Journal of Agriculture Food Systems and Community Development. 2022;11(2):1-26.

69.	Daigle M. Tracing the terrain of Indigenous food <i>sovereignties</i>. Journal of Peasant Studies. 2019;46(2):297-315.

70.	Whyte K. Food sovereignty, justice and indigenous peoples: an essay on settler colonialism and collective continuance. Whyte, KP. 2017.

71.	Hoover E. "You Can't Say You're Sovereign if You Can't Feed Yourself": Defining and Enacting Food Sovereignty in American Indian Community Gardening. American Indian Culture and Research Journal. 2017;41(3):31-70.
